# Exploring Zebrafish Larvae as a COVID-19 Model: Probable Abortive SARS-CoV-2 Replication in the Swim Bladder

**DOI:** 10.3389/fcimb.2022.790851

**Published:** 2022-03-11

**Authors:** Valerio Laghi, Veronica Rezelj, Laurent Boucontet, Maxence Frétaud, Bruno Da Costa, Pierre Boudinot, Irene Salinas, Georges Lutfalla, Marco Vignuzzi, Jean-Pierre Levraud

**Affiliations:** ^1^Institut Pasteur, Centre National de la Recherche Scientifique (CNRS) Unité mixte de Recherche (UMR) 3637, Unité Macrophages et Développement de l’Immunité, Paris, France; ^2^Institut Pasteur, Unité Populations Virales et Pathogénèse, Institut Pasteur, Paris, France; ^3^Université Paris-Saclay, Institut National pour la Recherche pour l’Agriculture, l’Alimentation et l’Environnement (INRAE), Université Versailles Saint-Quentin (UVSQ), Virologie et Immunologie Moléculaire (VIM), Jouy-en-Josas, France; ^4^Department of Biology, University of New Mexico, Albuquerque, NM, United States; ^5^Laboratory of Pathogen-Host Interactions (LPHI), Centre National de la Recherche Scientifique (CNRS), Université de Montpellier, Montpellier, France; ^6^Université Paris-Saclay, Centre National de la Recherche Scientifique (CNRS), Institut Pasteur, Institut des Neurosciences Paris-Saclay, Gif-sur-Yvette, France

**Keywords:** SARS-CoV-2, zebrafish, animal model, swim bladder, type I interferon

## Abstract

Animal models are essential to understanding COVID-19 pathophysiology and for preclinical assessment of drugs and other therapeutic or prophylactic interventions. We explored the small, cheap, and transparent zebrafish larva as a potential host for SARS-CoV-2. Bath exposure, as well as microinjection in the coelom, pericardium, brain ventricle, or bloodstream, resulted in a rapid decrease of SARS-CoV-2 RNA in wild-type larvae. However, when the virus was inoculated in the swim bladder, viral RNA stabilized after 24 h. By immunohistochemistry, epithelial cells containing SARS-CoV-2 nucleoprotein were observed in the swim bladder wall. Our data suggest an abortive infection of the swim bladder. In some animals, several variants of concern were also tested with no evidence of increased infectivity in our model. Low infectivity of SARS-CoV-2 in zebrafish larvae was not due to the host type I interferon response, as comparable viral loads were detected in type I interferon-deficient animals. A mosaic overexpression of human ACE2 was not sufficient to increase SARS-CoV-2 infectivity in zebrafish embryos or in fish cells *in vitro*. In conclusion, wild-type zebrafish larvae appear mostly non-permissive to SARS-CoV-2, except in the swim bladder, an aerial organ sharing similarities with the mammalian lung.

## Introduction

The COVID-19 pandemic has taken an enormous toll worldwide, in both human and economic losses. Although vaccination is finally under way, the SARS-CoV-2 virus is predicted to persist for years (Moore et al., 2021), and its variants represent an unpredictable threat. Thus, it will be necessary to continue the research efforts to understand its heterogeneous pathology and develop new drugs and vaccines.

Animal models play a central role during any pandemic since they are essential to analyzing pathology, transmission, and test vaccines and drugs. Besides non-human primates, other mammals such as Syrian hamsters and ferrets are naturally susceptible to SARS-CoV-2 (Muñoz-Fontela et al., 2020). Mice, the most widely used model for host–pathogen studies, require a transgene-mediated expression of human angiotensin-converting enzyme 2 (hACE2) to be infected (Lutz et al., 2020), although some recent variants replicate to a significant extent in wild-type mice (Montagutelli et al., 2021). All these models have several advantages and disadvantages. Non-human primates are very expensive, require large animal facilities, and are not conducive to large-scale experiments. hACE2 transgenic mice remain expensive and not readily available. As a result, expanding the repertoire of animal models for any disease is always beneficial and each model may shed light to unique aspects of the pathogen–host interaction. Here, we test if zebrafish larvae can be added to the list of suitable animal models for the study of COVID-19.

The zebrafish larva is an increasingly popular model to understand host–pathogen interactions (Torraca and Mostowy, 2018). Low cost of husbandry, high fecundity, and small size and transparency at early stages are among its main advantages. Thus, zebrafish larvae allow live imaging of pathogen dissemination at the whole organism to subcellular scales, and *in vivo* molecule screens in 96-well formats. Zebrafish is also a genetically tractable model, and thousands of mutant and reporter transgenic lines are available in fish facilities and repositories worldwide. Given that 80% of disease-associated genes of humans have a zebrafish ortholog (Howe et al., 2013), it is not surprising that zebrafish continue to be developed as models for human pathogens. Further, zebrafish is a bony vertebrate with an immune system that is also highly similar to ours. For instance, orthologs of the classical inflammatory cytokines (IL1β, TNFα, IL-6) as well as type I interferons (IFNs) are all found in zebrafish (Zou and Secombes, 2016). Interestingly, zebrafish adaptive immunity develops only at the juvenile stage, weeks after hatching (Lam et al., 2004), and the larva thus constitutes a system where innate immunity can be evaluated independently of adaptive responses. These assets make the zebrafish highly suitable to the study of host–virus interactions (Levraud et al., 2014).

Experimental infection has been established with various human viruses in zebrafish, including Herpes simplex virus 1 (Burgos et al., 2008), Chikungunya virus (CHIKV) (Palha et al., 2013), influenza A virus (IAV) (Gabor et al., 2014), and norovirus (Van Dycke et al., 2019). The upper temperature limit of proper zebrafish development, 33°C (Kimmel et al., 1995), may be an issue for some viruses; however, it corresponds to that of upper airways, and in fact SARS-CoV-2 replicates better at 33°C than at 37°C (V’kovski et al., 2021). The absence of lungs is another drawback to model a respiratory infection; however, teleost fish do possess an air-filled organ, the swim bladder, used for buoyancy regulation. Lungs of tetrapods and swim bladders of fish are evolutionarily related and share important structural homologies, such as surfactant coating (Cass et al., 2013). In support, inoculation of IAV in swim bladder resulted in localized infection (Gabor et al., 2014).

The zebrafish genome contains a unique, unambiguous ortholog of the gene encoding ACE2, the SARS-CoV-2 receptor; however, modest conservation of amino acids at the binding interface makes fish ACE2 proteins unlikely to bind the virus spike efficiently (Damas et al., 2020). Despite these *in silico* predictions, host susceptibility requires experimental validations, especially given that many other receptors and co-receptors for SARS-CoV-2 have been identified (Zamorano Cuervo and Grandvaux, 2020). In zebrafish larvae, based on single-cell transcriptomics, *ace2* is strongly expressed in a subtype of enterocytes (Postlethwait et al., 2021); the gut is also the organ with strongest *ace2* expression in humans.

There have been reports of the use of zebrafish to study COVID-19. We have recently reported pathological effects after exposure of zebrafish to recombinant SARS-CoV-2 spike protein, including accelerated heartbeat in larvae and severe olfactory damage causing transient hyposmia in adults after intranasal administration (Kraus et al., 2020). Injection of recombinant spike to adults has also been reported to induce adverse effects (Ventura Fernandes et al., 2020). Xenotransplantation of human lung cells in the swim bladder of adult zebrafish has been proposed to test the effect of an herbal drug on SARS-CoV-2 (Balkrishna et al., 2020). However, to date, no in-depth assessment of the ability of SARS-CoV-2 to replicate in zebrafish has been published.

Here we tested several tactics to infect zebrafish larvae with SARS-CoV-2, including bath exposure and microinjection in various organs or cavities. No evidence for production of new virions was obtained, but the distinct viral RNA dynamics obtained after swim bladder injection, together with immunohistochemical data, suggest that abortive infection occurred in that organ. Preventing type I IFN responses did not result in increased replication, consistent with the fact that SARS-CoV-2 inoculation did not result in strong IFN responses or induction of inflammatory cytokines.

## Results

### SARS-CoV-2 Replicates in Zebrafish Larvae Only When Injected in the Swim Bladder

**Figure 1:**
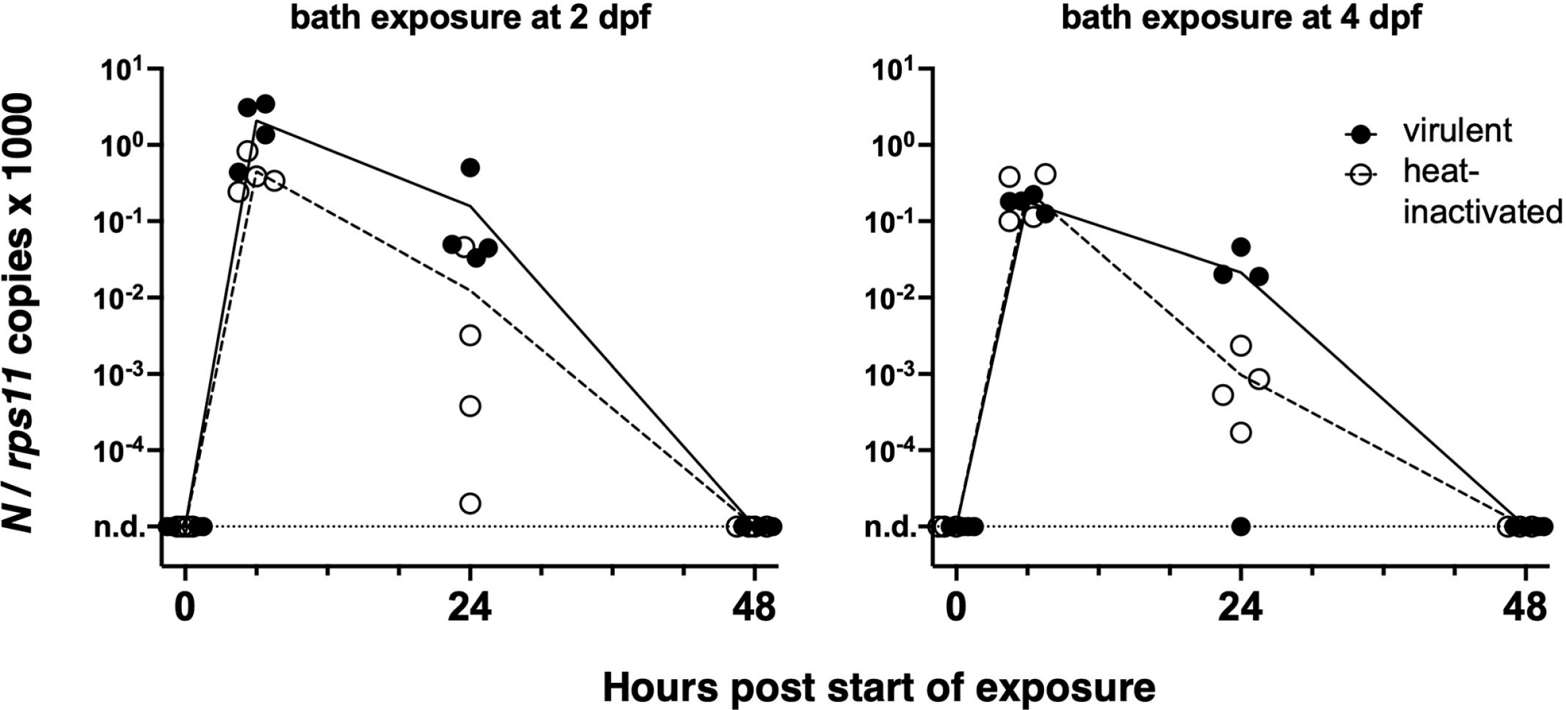
Bath exposure of zebrafish larvae to SARS-CoV-2. Kinetics of qRT-PCR measurements of polyadenylated viral N copies; each point corresponds to an individual larva. n.d., not detected.

We first tested if an early strain of SARS-CoV-2 would replicate in wild-type zebrafish larvae after bath exposure. We exposed 4-day post-fertilization (dpf) larvae with inflated swim bladders (ensuring an open gut) as well as 2-dpf dechorionated embryos with suspension of either live or heat-inactivated virus added to water (8 × 10^4^ PFU/ml). Larvae were then incubated at 32°C and observed regularly; no specific signs of distress were noted. After RNA extraction, the amount of polyadenylated SARS-CoV-2 *N* transcripts was measured by qRT-PCR. Although viral RNA was readily detectable after 6 h of exposure, it then declined and became undetectable after 48 h (**Figure 1**). Therefore, bath exposure failed to achieve infection.

**Figure 2:**
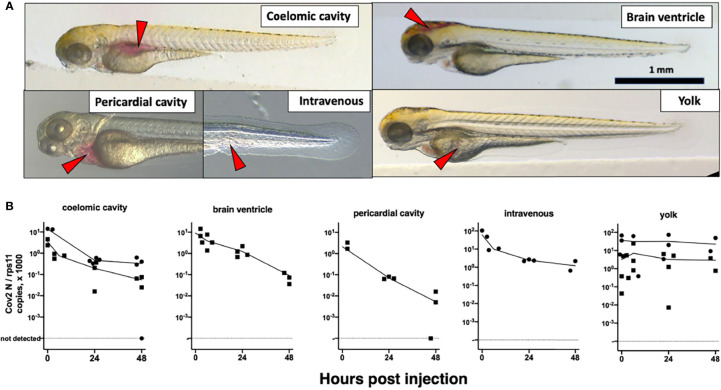
Microinjection of SARS-CoV-2 to 3-dpf wild-type larvae. **(A)** Illustrations of the targeted sites. Images taken less than 1 min after injection of the phenol red-colored SARS-CoV-2 suspension. Red arrowheads point to the sites of microinjection. **(B)** Quantification of polyadenylated N transcripts over time, assessed by qRT-PCR; each symbol is an individual larva. Circles and squares correspond to injection of viral suspensions 1 and 2, as labeled on **Table 1**, respectively. Lines connect the means of values measured at each time point.

We then turned to microinjection of larvae with SARS-CoV-2. Using a camera-fitted macroscope under a biosafety hood, a concentrated SARS-CoV-2 suspension was microinjected in various sites of 3 dpf larvae (**Figure 2A**). Compared with our previous experience of microinjection using the eyepieces of a stereomicroscope, this was significantly harder, notably due to lack of stereovision. These challenging injection conditions resulted in variability during early attempts; this later improved greatly, and although success of intravenous (IV) injections remained difficult to ascertain, others, notably in the coelomic cavity, were achieved reliably and in a reasonable time frame. Injection of the syncytial yolk cell was relatively easy, but leakage was often observed after capillary withdrawal, in which case larvae were discarded. Injected larvae were immediately rinsed and transferred into individual wells of 24-well plates, which were then incubated at 32°C. Larvae were imaged daily; none of the typical disease signs that we noted during other viral infections (e.g., edemas, spine bending, necrotic spots, slow blood flow) (Palha et al., 2013) were observed.

At various time points, individual larvae were euthanized and RNA extracted. Sense transcripts of SARS-CoV-2, genomic or subgenomic, are polyadenylated (Kim et al., 2020) and were measured after reverse transcription with a poly(dT) primer. The initial inoculum, measured in larvae lysed ~30 min postinjection (pi), was readily detectable by qRT-PCR (**Table 1**). Absolute quantification by qRT-PCR, using certified commercial reagents, revealed an amount of polyadenylated SARS-CoV-2 *N* transcripts that was ~10^4^-fold higher than the injected number of PFU (**Table 1**). Therefore, the overwhelming bulk of viral RNA injected in larvae must correspond to non-infectious molecules.

**Table 1:** Initial sense N copy numbers.

	Viral suspension 1	Viral suspension 2
Titer (PFU/mL)	1.13 × 10^8^	1.6 × 10^7^
PFU in a 2-nl inoculum	205	29
Median *N* copies measured in a cDNA sample corresponding to 1/100^th^ of larval extract	11,026	5,679
95% confidence interval	5,175–12,255	4,967–7,854
Number of samples	23	12
Ratio of median *N* copies to PFU	5,378	19,583

Quantification by RT-qPCR of polyadenylated viral N transcripts in zebrafish larvae microinjected with 2 nl of viral suspension (diluted 1.1-fold by addition of phenol red) in the coelomic cavity less than 1 h before lysis.

We then measured polyadenylated *N* copies over time. A decline was observed for all injection sites, with the notable exception of the yolk (**Figure 2B**). Amounts measured in yolk were highly variable at early time points, more than in other sites, probably due to leakage.

At this point, we wondered if this absence of replication may be due to a loss of infectivity of the virus in our microinjection conditions. We re-titered on Vero cells the leftover inoculum of one of our experiments, which had been left at room temperature for 1.5 h after thawing and addition of phenol red, and refrozen. The titer measured on Vero cells was 4.7 × 10^7^ PFU/ml, i.e., a ~2.5-fold drop from the original titer of 1.13 × 10^8^ PFU/ml. This was consistent with the drop expected from two rounds of freeze-thaw, suggesting that virus infectivity was not significantly decreased by our experimental conditions and confirming that we were injecting a significant amount of infectious particles.

To determine if the relatively high amounts detected in yolk at late time points were due to viral replication, we reanalyzed these RNA samples by performing reverse transcription with a primer that hybridizes to the 5′ leader sequence of negative-strand subgenomic RNAs, a hallmark of active SARS-CoV-2 replication (Kim et al., 2020; Wölfel et al., 2020). Antisense transcripts are expected to be less abundant than sense transcripts in infected cells and absent from virions. Such transcripts were detected in the initial inoculum but in lower amounts than polyadenylated transcripts (median values of 1,042 and 191 copies for coelom-injected larvae with viral suspensions 1 and 2, respectively). In coelom-injected larvae, these antisense transcripts decreased and became undetectable at 48 h postinjection (hpi). By contrast, in yolk-injected larvae, levels were stable (**Figure S1A**). Therefore, both sense and antisense viral RNA molecules appeared to be protected from degradation in the yolk, and there was no clear evidence for viral replication. Notably, we did not observe yolk opacity in injected animals, a hallmark of yolk cell infection with other viruses such as CHIKV (Palha et al., 2013) and Sindbis virus (SINV) (**Figure S2**).

**Figure 3:**
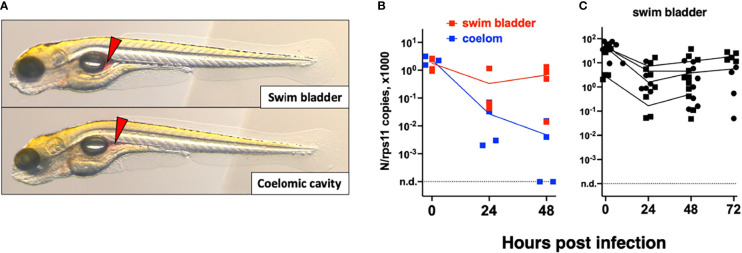
Microinjection of SARS-CoV-2 to 4-dpf larvae. **(A)** Illustrations of injection in the posterior end of the swim bladder or in the coelomic cavity. **(B, C)** Quantification of polyadenylated N transcripts over time, assessed by qRT-PCR; each symbol is an individual larva. **(B)** Comparison of swim bladder (red) and coelom (blue) injection in a single experiment. **(C)** Four more swim bladder injection experiments. Lines connect the means of values measured at each time point. Circles and squares correspond to injection of viral suspensions 1 and 2, as labeled on **Table 1**.

We then tested microinjection of SARS-CoV-2 in the swim bladder (SB), which inflates at 3.5–4 dpf (Parichy et al., 2009). We noticed that when the liquid was injected at the rostral end of the bladder, it was rapidly expelled *via* the pneumatic duct connecting the SB to the esophagus. By contrast, when liquid accumulated at the caudal end of the SB, if was well retained (**Figure 3A**). Therefore, injections were performed at 4 dpf by targeting the caudal half of the bladder; larvae with liquid injected at the rostral pole were discarded. As age-matched controls, we also injected 4-dpf larvae in the coelomic cavity, i.e., just next to, but outside of, the SB (**Figure 3A**).

Remarkably, in SB-infected larvae, after an initial decrease of viral transcripts during the first 24 h, their levels stabilized from 24 to 48 h; in contrast, the decline continued in coelom-injected larvae (**Figure 3B**). However, no disease signs were observed. We repeated the SB injection several times finding consistent results with a repeated trend of small re-increase from 24 to 48 h; extending the experiment by 1 day yielded comparable results at days 2 and 3 (**Figure 3C**). We also measured antisense transcripts in these larvae, observing the same trend (**Figure S1B**).

**Figure 4:**
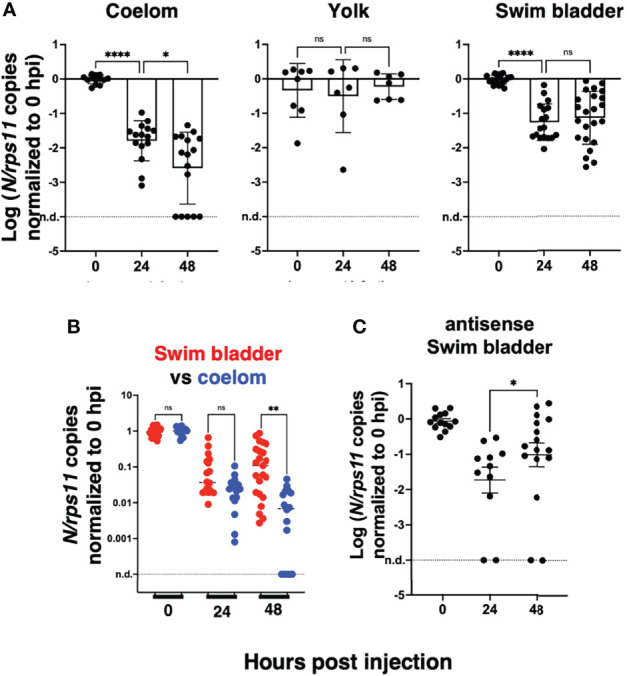
Statistical analysis of viral transcript quantifications, after normalization to the means of values measured at 0 hpi for each experiment. **(A)** Comparison of SARS-CoV-2 RNA loads over time in each microinjection location; ANOVA analysis of log-transformed values, not assuming equal SDs (Brown–Forsythe test with Dunn’s correction). Results pooled from four, two, and five experiments for coelom, yolk, and swim bladder injections, respectively. **(B)** Comparison of coelom and swim bladder injections at each time point; non-parametric multiple comparisons of non-transformed values (Kruskal–Wallis test with Dunn’s correction). **(C)** Comparison of antisense transcripts in the swim bladder, log-transformed values, Mann–Whitney test, pooled from three experiments. ns, not significant; *p < 0.05; **p < 0.01; ****p < 0.0001.

To perform statistical analysis with reasonable power, we normalized the results of each independent experiment to the mean of the values measured just after inoculation and then pooled the results by injection type. Because the dispersion increased considerably with time, we performed tests that allowed for unequal SDs when comparing time points. This analysis confirmed that after injection in the coelomic cavity, viral RNA amounts decline from 0 to 24 hpi and again from 24 to 48 hpi. By contrast, values measured in yolk were stable. In the SB, a very significant decrease is observed during the first 24 hpi, while from 24 to 48 hpi, a non-significant re-increase of the means is observed (**Figure 4A**). Comparison between the coelom and the SB showed a significantly higher level of viral RNA in the latter at 48 (but not 24) hpi (**Figure 4B**). These results clearly establish that while continuous viral RNA degradation occurs in the coelomic cavity, different events take place in the SB, which can be interpreted in several ways. One possibility could be that after 24 h of rapid decay of viral RNAs in the SB, this degradative reaction stops. Alternatively, *de novo* production could be taking place as a consequence of successful or abortive infection, compensating for ongoing degradation but not at a sufficient level to result in a detectable increase. Since antisense RNA are the first viral RNA species produced during the viral cycle, we analyzed the pooled normalized qPCR measurements of the antisense viral transcripts and found a significant increase of these transcripts from 24 to 48 hpi (**Figure 4C**). This increase of antisense but stagnation of sense viral RNAs suggests that some cells of the SB were invaded by SARS-CoV-2 but that the viral replication cycle was incomplete, resulting in abortive infection.

**Figure 5:**
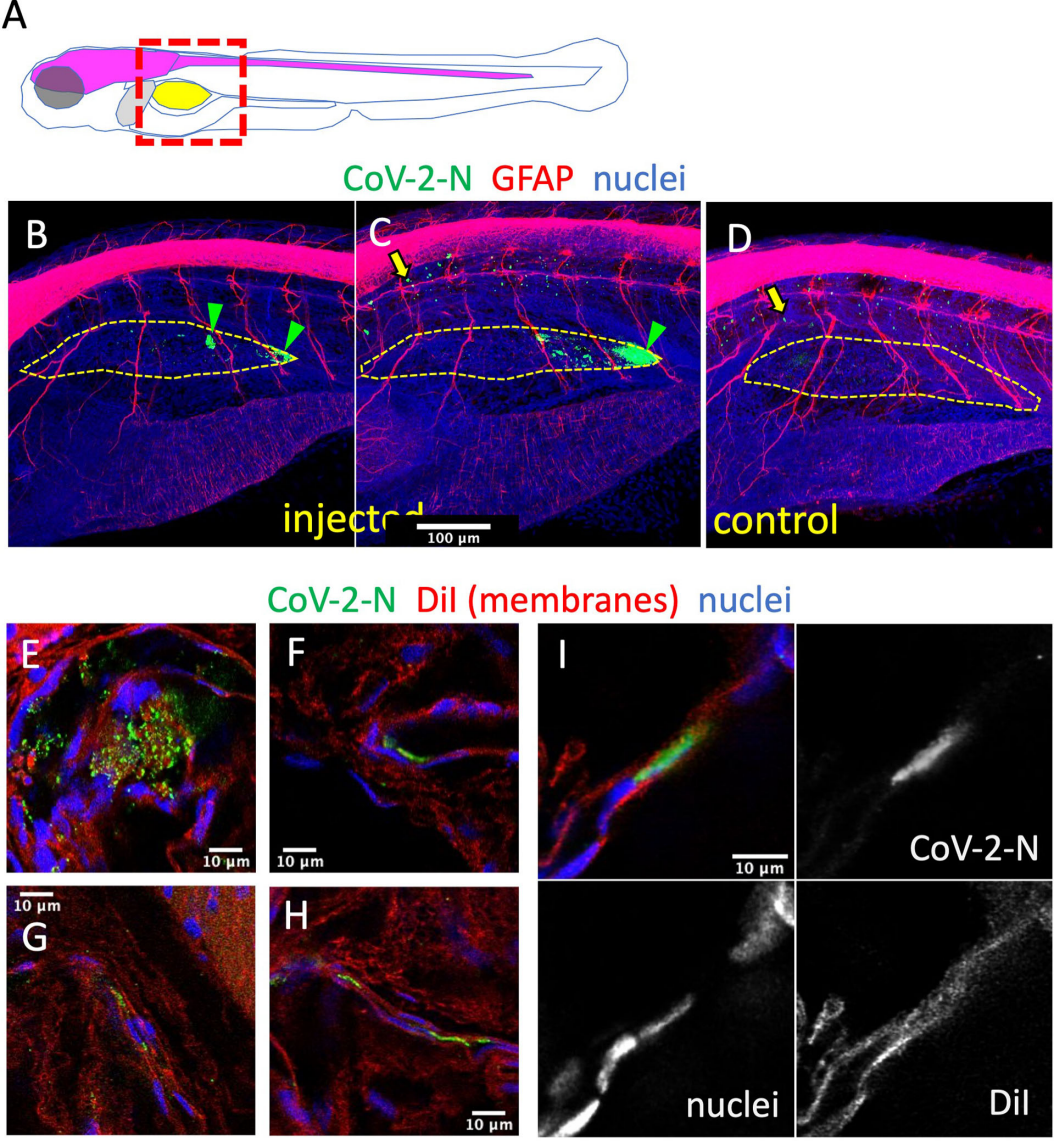
Immunodetection of infected cells in the swim bladder. **(A)** Scheme of the imaged region: the swim bladder is shown in yellow, the brain and spinal cord in magenta, the liver in gray. **(B–F)** Confocal images of SARS-CoV-2-injected **(B, C, E–I)** or uninjected **(D)** larvae fixed at 2 dpi and subjected to whole-mount immunohistochemistry with an anti-CoV-2-N antibody (green), an anti-GFAP antibody (red in **B–D**) or membranes stained by DiI (red in **E–I**), and stained nuclei (blue). **(B–D)** Maximal projections. The approximate contours of the partially collapsed swim bladders are shown with a dotted yellow line. N-positive cells shown with green arrowheads. Yellow arrows point to non-specific punctate signal in the notochord. **(E–I)** Single confocal planes.

To confirm infection by SARS-CoV-2 after SB injection, we used whole-mount immunohistochemistry (WIHC). We tested several commercial Abs against the SARS-CoV-2 nucleoprotein and selected a mouse Mab with minimal non-specific staining of naïve larvae, except for dots in the notochord that we routinely observe and which are due to the secondary antibody only (Levraud et al., 2009). As an anatomical reference, we also labeled glial fibrillary acidic protein (GFAP), to reveal glial cells and main nerves. In most virus-inoculated larvae at 2 dpi, a patchy signal for N could be clearly detected in the SBs which were partially collapsed due to the fixation and staining procedure (**Figures 5B–D**). 3D reconstruction (**Supplementary Video S1**) suggests that these signals correspond to a few infected cells in the bladder wall, generally located close to the rear pole. The signal was detected in 5/7 virus-injected larvae, and absent in 3/3 controls, but since this number was too low for statistical analysis, the experiment with repeated (without the GFAP stain) with a larger number of replicates (**Figure S3**) and at a higher resolution. A positive signal was detected in 11/14 samples, and only 1/14 among negative controls, with a highly significant statistical difference (p = 0.0003, Fisher’s exact test), based on a blind assessment by a naïve observer. A 3D reconstruction of these samples confirmed localization of signal to the collapsed SB walls (**Supplementary Video S2**), and their relationship with nuclear staining, as visualized on confocal slices, was consistent with N protein in the cytoplasm of at least some cells (**Supplementary Video S3**). To ensure that the signal was detected inside cells of the SB wall, we counterstained these samples with the lipophilic stain DiI and reimaged them. When the anti-N-labeled structures were parallel to the imaging plane, numerous DiI-stained spheroids were observed to be scattered between the N-containing spots (**Figure 5E** and **Supplementary Video S4**). This is reminiscent of the formation of vesicular replication factories in SARS-CoV-2-infected cells (Eymieux et al., 2021), but because of the lower resolution in the Z-axis, overlying and underlying plasma membranes were ambiguous in these cases. However, by imaging bladder walls where they are perpendicular or strongly oblique to the imaging plane, it was possible to determine the relative positions of N-positive patches and plasma membranes. Some of these patches appeared to be affixed to the bladder wall without a membrane separating them from the lumen (**Figure 5F** and **Supplementary Video S4**); however, others were clearly surrounded by membranes (**Figures 5G–I** and **Supplementary Videos S4**, **S5**) and were therefore localized within the cytoplasm of swim bladder epithelial cells.

### Variants of Concern Do Not Show Increased Infectivity in Wild-Type Larvae

**Figure 6:**
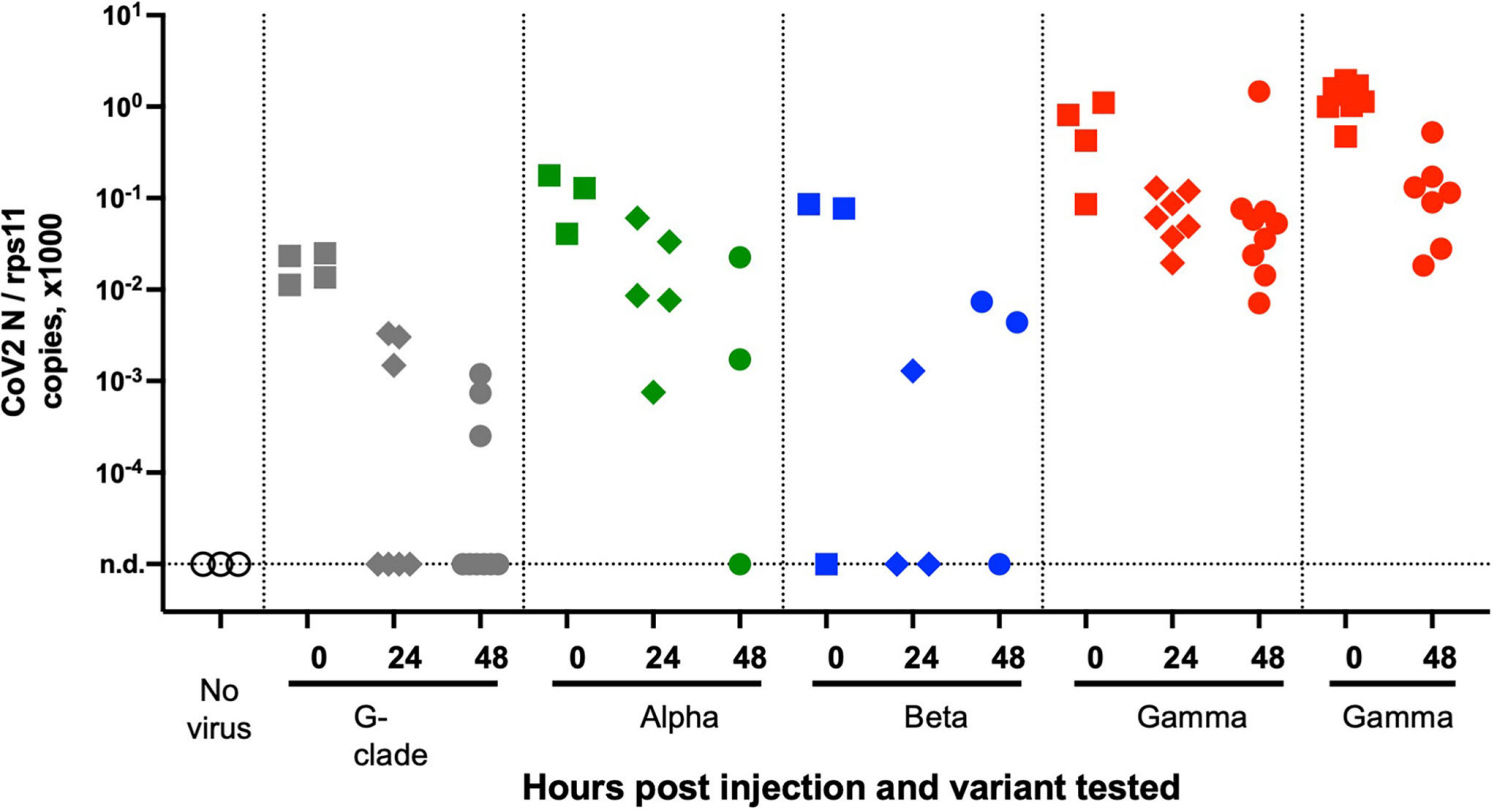
Testing SARS-CoV-2 variants. qRT-PCR analysis of larvae at various times after injection of 2 nl of virus suspension in the swim bladder. Dotted lines separate independent experiments.

We then tested a series of SARS-CoV-2 variants by SB inoculation. We obtained aliquotes from early passages after isolation of clinical strains, which had been titered at 3.10^7^ PFU/ml or more and thus did not require further concentration. We tested the alpha variant (formerly known as UK variant, or B1.1.7), the beta variant (South African variant, B1.351), and the gamma variant (Brazilian variant, P1) as well as a representative of the G-clade which arose early during the pandemic. Non-diluted viral suspensions were injected as described above in the SB of 4-dpf larvae and were then monitored for 2 days; no clinical signs were observed. Viral replication was assessed by qRT-PCR. A global decline of polyadenylated N transcripts over time was observed with all variants (**Figure 6**). One unique larva injected with the gamma variant was found to contain slightly more N copies than the initial inoculum; therefore, the experiment was repeated for the gamma variant, and again, one larva did not show the same decline as others. Thus, results obtained with the gamma variant were comparable to those obtained with the initial strain, with a fraction of larvae in which some replication appeared to take place. No replication was found with the other strains, which also corresponded to lower inocula according to qPCR results. Overall, we saw no evidence for an increased infectivity of SARS-CoV-2 variants in zebrafish larvae.

### A Defective Type I Interferon Response Does Not Increase SARS-CoV-2 Replication

Type I interferons (IFNs) are key antiviral cytokines in vertebrates, including teleost fish. We thus tested if SARS-CoV-2 may replicate in larvae with a crippled type I IFN response.

**Figure 7:**
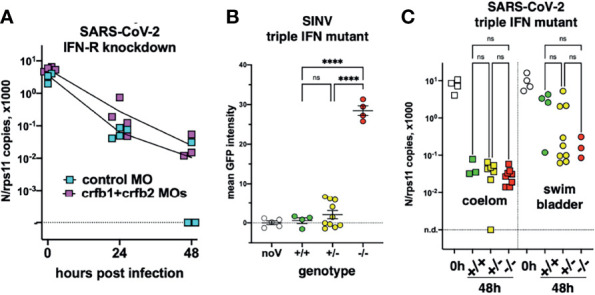
Viral infection in IFN-defective larvae. **(A)** IFN-receptor (crfb1 and crfb2 genes) or control morphants infected at 3 dpf in the coelomic cavity; qRT-PCR. **(B, C)** Offspring from an incross of heterozygous triple IFN-mutants. **(B)** Larvae injected with SINV-GFP IV at 3 dpf, analyzed by fluorescence imaging at 48 hpi. **(C)** Larvae injected with SARS-CoV-2, either at 3 dpf in the coelom or at 4 dpf in the swim bladder; analyzed by qRT-PCR at 0 or 48 hpi. Statistical analysis by ordinary 1-way ANOVA in **(B)**, by Kruskal–Wallis test in **(C)**. ns, not significant; ****p < 0.0001.

First, we used morpholino-mediated knockdown of the type I IFN receptor chains CRFB1 and CRFB2, known to make zebrafish larvae hypersusceptible to infection with CHIKV or SINV (Palha et al., 2013; Boucontet et al., 2018). After injection of SARS-CoV-2 in the coelom of 3-dpf larvae, decline of N transcripts was found to be similar in IFNR-knocked-down larvae than in controls (**Figure 7A**).

To ensure a long-lasting suppression of the IFN response, we used a newly generated mutant zebrafish line dubbed “triple ϕ,” in which the three type I IFN genes *ifnphi1*, *ifnphi*2, and *ifnphi*3, tandemly located on chromosome 3, have been inactivated by CRISPR. Heterozygous triple ϕ mutants were viable and fertile; incrossing them yielded homozygous embryos at the expected Mendelian ratio of ~25%. Homozygous triple ϕ mutants could be raised up to juvenile stage, but, unlike their siblings, died in the 2 weeks following genotyping by fin clipping. To validate the phenotype of the mutants, we injected SINV-GFP to 3-dpf larvae from a heterozygous incross. 48 h later, all larvae were alive although some showed strong signs of disease, including loss of reaction to touch, abnormal heartbeat, slow blood flow, edemas, and opacified yolk spots. All larvae were imaged with a fluorescence microscope to measure the extent of infection, then lysed individually and genotyped. The homozygous mutants displayed a considerably higher level of fluorescence (**Figure 7B**) and were also identified *a posteriori* as the sickest larvae, confirming that triple ϕ mutants are hypersusceptible to viral infection.

Larvae from triple ϕ heterozygous incrosses were thus injected with SARS-CoV-2, either in the coelomic cavity at 3 dpf or in the SB at 4 dpf. Larvae were lysed at 48 hpi, analyzed by qRT-PCR, and genotyped. Consistent with previous results, a 100-fold decrease of viral RNA was observed in coelom-injected larvae, while a weaker decrease was observed for SB injection, with a bimodal distribution suggesting that infection happened in about one-third of cases. In both situations, viral loads in homozygous triple ϕ mutants were not different from their wild-type siblings (**Figure 7C**). Thus, our results indicate that type I IFN responses are not responsible for the lack of replication of SARS-CoV-2 observed in wild-type zebrafish larvae.

### Lack of Detectable Inflammatory Responses in SARS-CoV-2-Injected Larvae

**Figure 8:**
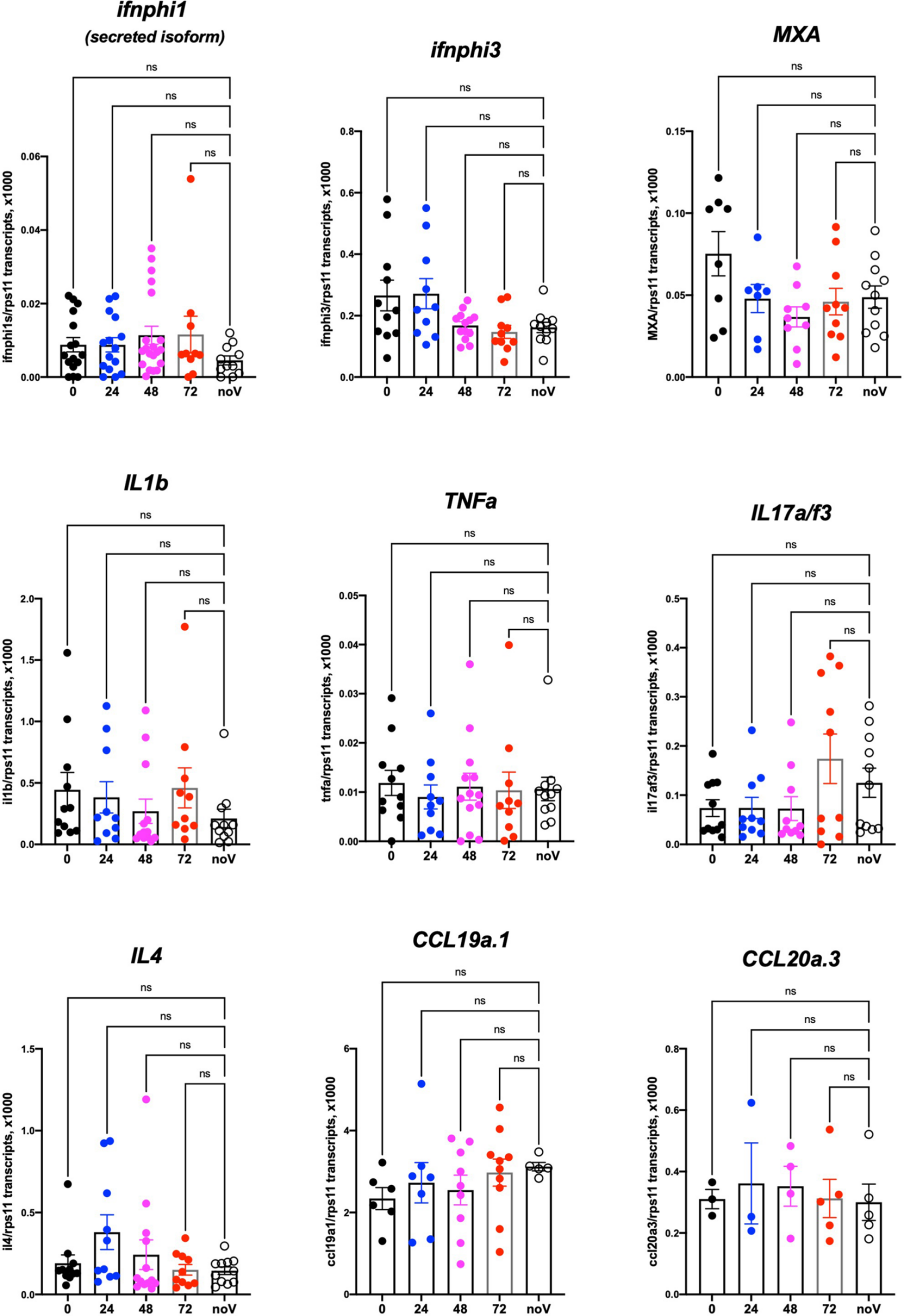
Host response after SARS-CoV-2 injection in the swim bladder. qRT-PCR, pool of 4 independent experiments (except for ccl19a.1 and ccl20a.3, 3 and 2 experiments respectively). Numbers on the X-axis refer to hours postinjection; noV (for “no Virus”): pooled uninjected negative controls, age-matched to 24, 48, or 72 hpi. One-way ANOVA analysis. ns, not significant.

We then tested if SARS-CoV-2 inoculation in the SB resulted in induction of a type I interferon response or inflammatory cytokines. For this, we performed qPCR on dT17-primed cDNAs from whole larvae. Based on our previous results (Levraud et al., 2019; Kraus et al., 2020), we tested the main type I interferon genes inducible in larvae, namely, *ifnphi1* and *ifnphi3*; the strongly IFN-inducible gene *MXA*; the classical inflammatory cytokines *il1b* and *tnfa*; cytokines that reflect induction of type 2 or type 3 responses, *il4* and *il17a/f3*, respectively; and chemokines *ccl19a.1* and *ccl20a.3*. Although individual experiments suggested significant induction of *ifnphi1* at 48 hpi or *il17a/f3* at 72 h, this could not be replicated; as shown on **Figure 8**, in which data from 4 independent experiments have been pooled, no significant change in expression of any of these genes can be observed compared to uninjected control larvae. Similar negative results were obtained with larvae injected at different sites (not shown).

Although these results do not exclude a local response to SARS-CoV-2, they are in striking contrast with those we obtained previously in larvae infected with other pathogens such as SINV or *Shigella flexneri*, for which many of these genes were induced more than 100-fold (Boucontet et al., 2018). Since these experiments had been performed at 28°C, we verified that zebrafish larvae are also able to mount a strong type I response at 32°C (**Figure S4**).

### Mosaic Overexpression of hACE2 Is Not Sufficient to Support SARS-CoV-2 Infection of 3-dpf Larvae or Fish Cells *In Vitro*

**Figure 9:**
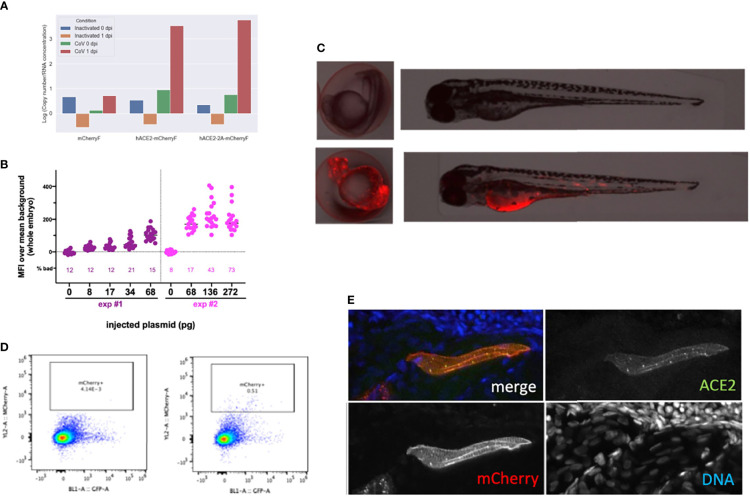
Overexpression of hACE2-mCherry by plasmid injection at the 1-cell stage. **(A)** Verification of construct functionality by transfection in BHK cells. N mRNA levels measured 24 h after exposure of transfected cells with inactivated or with active SARS-CoV-2. **(B)** Optimization of the plasmid dose. Fluorescence intensity measured in 24-hpf embryos after injection at the 1-cell stage of the specified amount of the pz26hACE2-mCherryF plasmid together I-SceI, for the 25% embryos with the best expression in each group. The percentage of misshapen embryos in each group is indicated on the bottom of the graph. **(C)** representative image of a 24-hpf embryo (left) and a 3-dpf larva (right) after mock injection (top) or injection of 68 pg of pz26hACE2-mCherryF. **(D)** Representative flow cytometry analysis of cells dissociated from 4-dpf larvae, mock-injected (left) or injected with 68 pg of pz26hACE2-mCherryF (right). **(E)** Immunohistochemistry of a larva injected with 68 pg of pz26hACE2-2A-mCherryF, showing ACE2 detection of a mCherry-positive muscle fiber.

Finally, we tested if mosaic overexpression of human ACE2 in zebrafish larvae would increase their infectivity of SARS-CoV-2. We subcloned the *hace2* ORF in fusion with mCherryF under the control of the promoter of the ubiquitous ribosomal protein RPS26. In addition, the fragment is flanked by two inverted I-SceI meganuclease sites for higher transgenesis efficiency (Grabher et al., 2004). In order to be sure that the in-frame fusion of hACE2 with mCherry would not interfere with SARS-CoV-2 binding to its receptor and entry in the target cells, another construct was done by inserting a self-cleaving 2A peptide between hACE2 and mCherry ORFs. Both constructs were tested in BHK cells and increased by ~100-fold their infection by SARS-CoV-2 (**Figure 9A**). We optimized the injected dose of plasmid; 68 pg was the amount yielding the highest mCherry expression without increasing the proportion of misshapen embryos (**Figure 9B**). In 24-hpf embryos, many mCherry^+^ cells, randomly distributed, were visible in these embryos under the fluorescence microscope. In swimming larvae, mCherry^+^ cells were still clearly visible but in lower amounts (**Figure 9C**). To get a quantitative assessment of their frequency, we dissociated 4-dpf larvae and analyzed the suspension by flow cytometry, which indicated that ~0.5% of the cells were mCherry^+^ (**Figure 9D**). Larvae were fixed and processed by immunohistochemistry, which confirmed ACE2 expression at the membrane of mCherry^+^ cells (**Figure 9E**).

**Figure 10:**
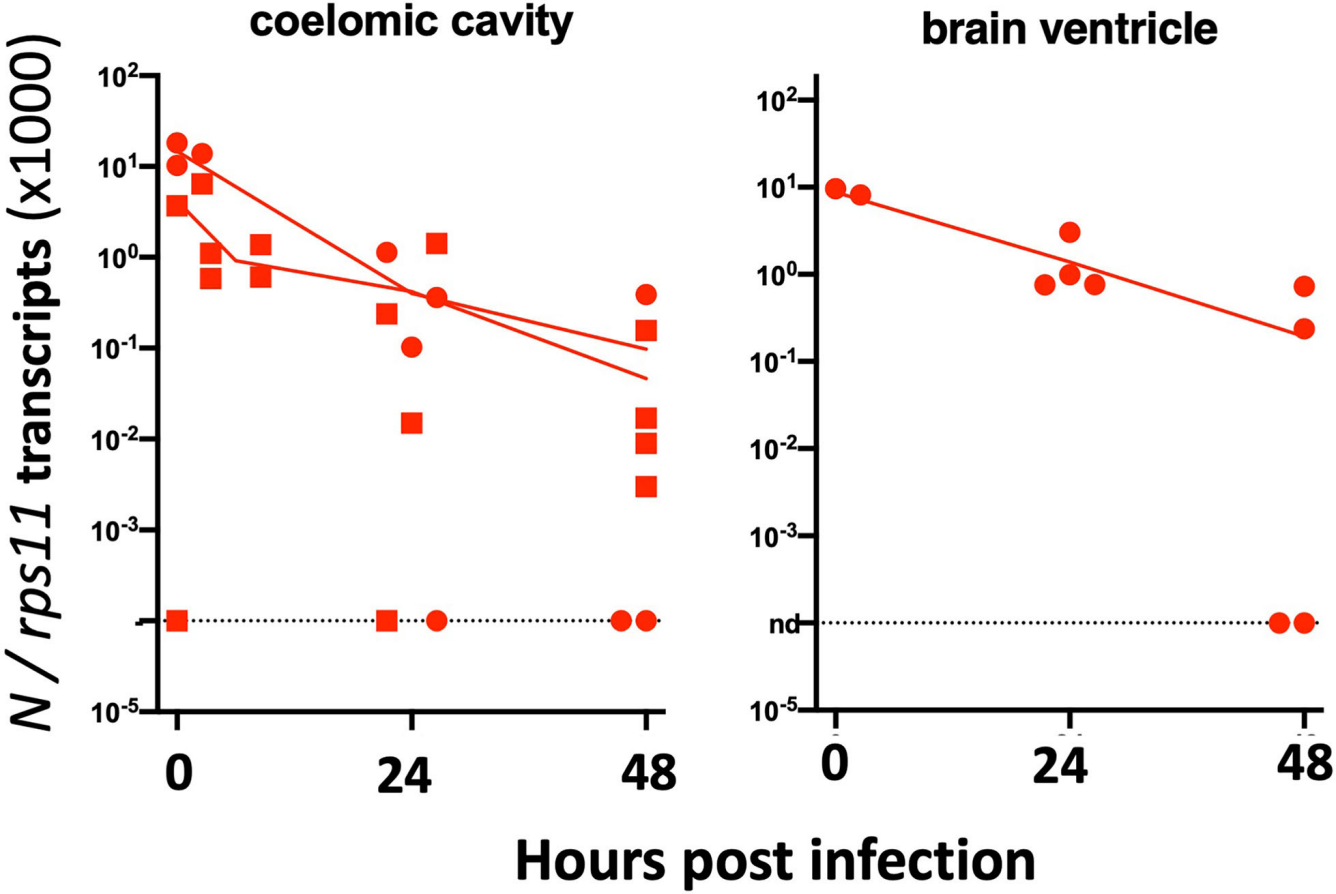
Injection of 3-dpf hACE2-mCherry mosaic larvae. Quantification of sense N transcripts in individual hACE2-mCherry mosaic larvae injected in coelomic cavity (left; one experiment with hACE2-mCherry, one with hACE2-2A-mCherry) or brain ventricle (right; with hACE2-2A-mCherry) by qRT-PCR.

Zebrafish AB eggs were injected with the plasmid, and at 3 dpf, the 25% larvae displaying the highest mCherry expression and good morphology were selected. They were then microinjected with SARS-CoV-2 in the coelom or the brain ventricle and processed as above. qRT-PCR analysis revealed that viral mRNA transcripts decreased just as it did in AB larvae (**Figure 10**). Thus, this approach did not increase the infectivity of SARS-CoV-2 in zebrafish larvae.

**Figure 11:**
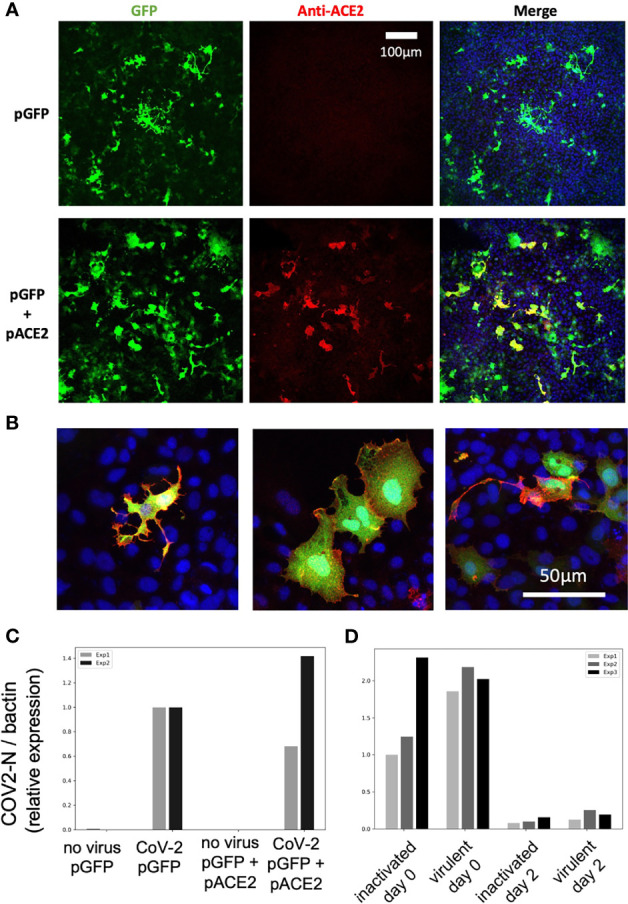
SARS-CoV-2 does not replicate on EPC cells transfected with human ACE2. **(A, B)** Confocal microscopic images of EPC cells cultures 3 days after transfection. **(A)** Assessment of transfection efficiency at low magnification. **(B)** Demonstration of hACE2 expression at the membrane of cells transfected with both plasmids, high magnification, merge images of GFP, hACE2, and nuclei labels. **(C, D)** qRT-PCR measurement of N copies in cells. **(C)** Measurement at 2 days postexposure with virulent SARS-CoV-2, comparison of GFP and GFP+hACE2 overexpression. **(D)** Decline of RNA levels from day 0 to day 2 postexposure, and comparison of heat-inactivated and virulent virus.

We finally tested if hACE2 overexpression by *in vitro* cultured fish cells made them susceptible to SARS-CoV-2, using the cyprinid cell line EPC. EPC cells were co-transfected with GFP and hACE2 expression plasmids; transfection efficiency and membrane hACE2 expression was verified by IHC (**Figures 11A, B****)**. These transfected cells were incubated with active or heat-killed SARS-CoV-2 at a MOI of 0.1 and then tested for viral replication by qRT-PCR on cell lysates. No difference was observed between GFP-only and GFP+hACE2-expressing cells (**Figure 11C**); furthermore, the amount of N transcripts fell dramatically from day 0 to day 2 (**Figure 11D**), showing that hACE2-expressing EPC cells were not able to support SARS-CoV-2 replication.

## Discussion

We report here our in-depth attempt to infect zebrafish larvae with SARS-CoV-2. Only larvae were tested because they present multiple practical advantages over adult fish: they can be rapidly generated in large quantities, can be incubated in multi-well plates, are highly amenable to imaging, and are subject to fewer ethical regulations; therefore, they would be most suitable to drug screening. Whether juvenile or adult zebrafish would be more susceptible to SARS-CoV-2 remains to be tested.

We used absolute qRT-PCR of viral transcripts to test for viral replication. Surprisingly high numbers were measured shortly after injection, as the concentrated viral suspensions we used contained a considerable amount of noninfectious viral molecules, including negative-strand species. In all likelihood, these molecules were released by infected Vero-E6 cells during the production of the virus stock, possibly by living cells as defective viral particles or in vesicles such as exosomes, or as free or membrane-bound RNA from dying cells. Whatever their origin, they complicate the detection of active viral replication, which has to generate enough molecules to exceed this background.

In almost all of our tests, a rapid (10- to 100-fold) decrease of mRNA copies was observed during the first day, likely due to degradation of noninfectious RNA species. After a few hours of bath exposure, viral RNA was detected in doubly rinsed larvae; this did not require active fusion or viral particles as RNA was also detected after exposure to heat-inactivated virus and may have resulted from sticking of particles to skin surfaces or entry in the pharyngeal cavity. Two days after the starting of exposure, viral RNA was undetectable and thus the virus failed to achieve infection by bath, consistent with the results of Kraus et al. (2020).

Microinjection is the most common way to infect zebrafish larvae with viruses (Levraud et al., 2014). After microinjection of a few nanoliters in larvae, the inoculum was readily measurable; however, when injected in the coelom, the pericardium, the bloodstream, or the brain ventricle, viral RNA copy numbers then steadily declined, indicating unsuccessful infection. Two injection sites yielded different results: the yolk and the swim bladder. In the yolk, no RNA decrease was observed, suggesting that viral RNA molecules—perhaps owing to their coating with nucleoprotein and/or their localization in vesicles—were spared from degradation. Importantly, the yolk was unique among all tested sites as the one where injection is performed inside the cytosol of a cell (the yolk syncytial cell, not to be confused with the yolk sac) and not in the extracellular milieu. This does not necessarily prevent infection, as other viruses, such as CHIKV (Briolat et al., 2014) or human noroviruses (Van Dycke et al., 2019), have been shown to infect larvae after yolk injection. No signs of yolk infection (such as opacity observed with CHIKV and SINV) were observed, and no increase of viral mRNA was observed, so we believe that yolk injection did not result in active SARS-CoV-2 replication.

By contrast, injections in the SB resulted in a ~20-fold decrease of mRNA copies during the first day, followed by a stabilization of sense viral RNA levels and a modest re-increase of antisense RNAs; this was accompanied by progressive dispersion of data points. This suggests that abortive infection occurred in some but not all larvae after SB infection. Because of the considerable spread in measured copy numbers at 2 dpi, the re-increase is statistically borderline, but the bimodal distribution observed in the independent type I IFN mutant assay, and the comparisons with injections in the coelom support this interpretation. Importantly, this was reinforced by an independent immunohistochemistry assay as we observed, in a fraction of injected larvae, a few cells in the SB wall labeled by an antibody that detects the SARS-CoV-2 nucleoprotein. By staining membranes, we confirmed the presence of this protein in the cytosol of cells in the bladder wall. Nucleoprotein-rich patches were also interspersed with numerous vesicular structures suggesting formation of replication factories. Thus, we believe that a few cells of the bladder wall were successfully invaded by SARS-CoV-2, although there is no evidence that this resulted in the production of new infectious virions. It also remains unclear why infections succeed in only a fraction of SB-injected larvae. This could be due to a very low effective inoculum, but this seems unlikely since the success rate was not obviously higher with viral suspension 1 than suspension 2, despite a 7-fold higher titer.

Unfortunately, given the small re-increases of N copy number observed, it can be predicted that even if successful virus replication had occurred, the amount of infectious particles remained too small to be detectable by direct titrations from infected whole larvae. This is a clear limitation of our study that we hope can be solved when conditions allowing stronger replication are identified.

The stabilization of the amount of sense viral RNA in the SB could also be interpreted as persistence rather than *de novo* production. In fact, these two possibilities are non-exclusive; however, the fact the during the first 24 h, the number of viral mRNA strongly decreases, indicates that the SB environment is not hospitable and it is unlikely that degradative mechanisms become less active as the fish age, which is usually accompanied by stronger immune reactions. Could internalization of virions by cells of the SB protect them from this activity? If the SB epithelium was scavenging the luminal content, then a regular distribution of N across the SB would be expected; instead, the patchiness observed in IHC experiments is consistent with the infection of very few cells followed by *de novo* production of N by these cells.

What could be the mechanisms mediating virus clearing from the SB? This has not been studied in the context of viral infection, but in a model of filamentous candidiasis, the recruitment of neutrophils to the SB lumen and the subsequent extracellular trap production (Gratacap et al., 2017) and of inflammatory macrophages (Archambault et al., 2019) have been observed. A strong expression of a defensin gene specifically in the SB has also been reported (Oehlers et al., 2011). It seems also plausible that microbes trapped in mucus may be cleared from the SB *via* the pneumatic duct, although such a mechanism has not been described to our knowledge.

It is interesting that the organ found to be most permissive to infection in zebrafish larvae is homologous to the human lung which is the primary target of the virus. We do not know if SB epithelial cells express *ace2*. Unfortunately, there is no “swim bladder epithelium” subset in the scRNAseq zebrafish developmental atlas (Farnsworth et al., 2020), perhaps because these cells are too rare or difficult to isolate enzymatically. However, the SB derives from the gut, which is the only organ in which cells highly express *ace2* in the atlas (Postlethwait et al., 2020). One may speculate that, besides surface protein expression, biophysical parameters such as surfactant coating or pressure-mediated tension of the epithelium could contribute to infectivity.

Given the expression of *ace2* in zebrafish enterocytes, it would also have been interesting to microinject the virus in the gut lumen. We tried, unsuccessfully, in part because of the close apposition of the gut and the easily damaged yolk. It should be noted, however, that coelomic injections (the equivalent of intraperitoneal injections), comparatively easy to perform, deliver the virus in close proximity to the basal side of enterocytes but do not yield successful infection.

Not surprisingly, the SARS-CoV-2 virus has evolved during the pandemic with successful waves of variants of concern with mutated spike protein, predicted to modulate binding to hACE2 and antibody neutralization. In the normally non-permissive wild-type mouse model, it has been shown that the beta and gamma variants replicated to a significant extent (Montagutelli et al., 2021). We tested several variants, including those two, in the zebrafish swim bladder model but did not find increased infectivity compared to the reference strain.

To stay within the thermal range of both virus and host, we incubated SARS-CoV-2-injected larvae at 32°C. Because SARS-CoV-2 replicates better at 33°C than at 37°C in mammalian cells (V’kovski et al., 2020) (and our own observations), this is unlikely to be the reason for the poor replication of the virus in larvae. We also verified that at this temperature, larvae are able to mount a type I IFN response against another virus, eliminating temperature stress as the explanation for the lack of inflammatory response of zebrafish larvae to SARS-CoV-2. This is more likely due to the small number of infected cells in our conditions and possibly also active inhibition of some innate immune pathways by the virus. Protocols resulting in stronger infection will be needed for studying SARS-CoV-2-induced inflammation in zebrafish larvae. This absence of measurable type I IFN response is consistent with the finding that IFN or IFN-R deficiency did not rescue virus infectivity. Thus, a limited compatibility between the virus and the host, rather than an intrinsic active resistance, seems the most likely explanation for our largely negative results.

The mosaic overexpression of hACE2 did not result in infectivity of 3-dpf larvae by SARS-CoV-2. We do not know if this was due to the relatively small number of cells expressing the transgene (<1%), to low expression or misfolding of the hACE2 protein, and/or to other causes. As an alternative strategy, we also tested injection of synthetic mRNA encoding hACE2-mCherryF; this resulted in clear ubiquitous mCherry expression at 24 hpf, but it became undetectable by 2 dpf (not shown). This suggests that the hACE2 protein and mRNA have a relatively short half-life in the zebrafish larval context. This issue may be solved by the establishment of stable transgenic zebrafish lines expressing hACE2. However, we also tested the effect of overexpression of hACE2 in the more stable context using the EPC cell line. EPCs are derived from a cyprinid fish and used routinely to test the pro- or antiviral activity of zebrafish genes by overexpression [e.g., (Langevin et al., 2013)]. However, the expression of hACE2 was not sufficient to allow replication of SARS-CoV-2 on these cells. The lack of replication may be due to the need for co-expression of the transmembrane serine protease TMPRSS2, which has been shown to greatly increase SARS-CoV-2 infectivity (Hoffmann et al., 2020). We also attempted to overexpress human TMPRSS2 in zebrafish embryos, by either plasmid or mRNA injection; unfortunately, this was found to be highly toxic, as it resulted in severe developmental anomalies that precluded injections.

Thus, it seems that fish cells are intrinsically unable to support full SARS-CoV-2 replication, which could be due to the lack of a required host cell component that the virus must interact with, or to intrinsic immunity linked to the expression of a restriction factor, or both. Given the dissimilarities between human and zebrafish ACE2 in the Spike-interaction region (Kraus et al., 2020), a good receptor is probably missing, but we have shown that hACE2 overexpression is not sufficient. One interesting clue to a possible restriction mechanism may lie with the aspect of cells containing nucleoprotein observed in the SB by IHC, which contain numerous lipidic vesicles reminiscent of replication factories, albeit larger and less regular than those observed in Vero cells (Eymieux et al., 2021). This suggests an improper interaction of viral and host cell components required to properly establish these double-membrane structures.

In conclusion, our experiments indicate that the zebrafish larva is largely not infectable by SARS-CoV-2, except when the virus is injected in the swim bladder, which appears to result in abortive infection in a subset of the animals. Further optimization of infection procedures, starting with the generation of transgenic zebrafish expression stably expressing human ACE2, and identification of mechanisms that prevent SARS-CoV-2 replication in fish cells, will be needed to unleash the full potential of the zebrafish larva in the fight against COVID-19.

## Methods

### Ethical Statement

Animal experiments described in the present study were conducted according to European Union guidelines for handling of laboratory animals (http://ec.europa.eu/environment/chemicals/lab_animals/home_en.htm) and were approved by the Ethics Committee of Institut Pasteur.

### Fish

Wild-type zebrafish (AB strain), initially obtained from ZIRC (Eugene, OR, USA), were raised in the aquatic facility of Institut Pasteur. After natural spawning, eggs were collected, treated for 5 min with 0.03% bleach, rinsed twice, and incubated at 28°C in Petri dishes in Volvic mineral water supplemented with 0.3 µg/ml methylene blue (Sigma-Aldrich, St. Louis, Missouri, USA). After 24 h, the water was supplemented with 200 µM phenylthiourea (PTU, Sigma-Aldrich) to prevent pigmentation of larvae. After this step, incubation was conducted at 24°C, 28°C, or 32°C depending on the desired developmental speed. Developmental stages given in the text correspond to the 28.5°C reference (Kimmel et al., 1995). Sex of larvae is not yet determined at the time of experiments.

Triple type I interferon CRISPR mutants have been generated by the AMAGEN transgenesis platform (Gif-sur-Yvette, France) by co-injection of CAS9 with two sgRNA targeting *ifnphi1* (target sequence, GCTCTGCGTCTACTTGCGAAtgg) and *ifnphi2* (target sequence, ATGTGCGCGAAAAAGAGTGCtgg) in one-cell eggs from homozygous ifnphi3^ip7/ip7^-null mutants of the AB background (Maarifi et al., 2019). After growth to adulthood, a founder was identified that co-transmitted mutations in *ifnphi1* and *ifnphi2* in addition to the *ip7* mutation of *ifnphi3*. The *ip9* allele mutation in *ifnphi1* consists in a 7-base pair deletion in the first exon of the secreted isoform (GAATGGC, 75 bases downstream of the start ATG). The *ip10* allele in *ifnphi2* consists in a 19-bp deletion in the first exon (TGCGTTCTTATGTCCAGCA, 20 bases downstream of the start ATG). This founder was crossed with AB fish, and F1 fish triply heterozygous for mutations *ip7*, *ip9*, and *ip10* were selected to establish the “triple φ” mutant line. As expected, since *ifnphi1*, *ifnphi2*, and *ifnphi3* are closely located in tandem on a 35-kb region of zebrafish chromosome 3, the *ip7, ip9*, and *ip10* mutations were always found to co-segregate. Genotyping PCR primers are listed in **Table 2**.

**Table 2:** Primers used in this study.

Genotyping primers		5′–>3′ sequences	
Gene (allele)	ZFIN ID	Forward primer	Reverse primer
*ifnphi1 (wt)*	ZDB-GENE-030721-3	CTCTGCGTCTACTTGCGAAT	CTCCAACCCAACAAGTCGC
*ifnphi1 (ip9)*		AGCTCTGCGTCTACTTGCTT	CTCCAACCCAACAAGTCGC
*ifnphi2 (wt)*	ZDB-GENE-071128-1	TCTTGGGGATTCATGTCTTCA	GCGAAAAAGAGTGCTGGACA
*ifnphi2 (ip10)*		TCTTGGGGATTCATGTCTTCA	GTGCGCGAAAAAGAGACGAA
*ifnphi3 (wt)*	ZDB-GENE-071128-2	AGAATGGACCTTCACCGTGT	CGCAGTCTCCAGAAGTGTAT
*ifnphi3 (ip7)*		ATTCCGTATAGGCATCTGATT	CGCAGTCTCCAGAAGTGTAT
			
**RT primers**			
(dT)17		TTTTTTTTTTTTTTTTT	
sgLeadSARSCoV2-F		CGATCTCTTGTAGATCTGTTCTC	
			
**qPCR primers**			
*rps11*	ZDB-GENE-040426-2701	CGTGAAAGACTGTCTTCCGT	TCAACAACACAGAGGAGCCA
*ifnphi1*	ZDB-GENE-030721-3	TGAGAACTCAAATGTGGACCT	GTCCTCCACCTTTGACTTGT
*ifnphi3*	ZDB-GENE-071128-2	GAGGATCAGGTTACTGGTGT	GTTCATGATGCATGTGCTGTA
*mxa*	ZDB-GENE-030721-5	GACCGTCTCTGATGTGGTTA	GCATGCTTTAGACTCTGGCT
*tnfa*	ZDB-GENE-050317-1	TTCACGCTCCATAAGACCCA	CAGAGTTGTATCCACCTGTTA
*il1b*	ZDB-GENE-040702-2	GAGACAGACGGTGCTGTTTA	GTAAGACGGCACTGAATCCA
*il4*	ZDB-GENE-100204-1	GACAGGACACTACTCTAAGAA	CAGTTTCCAGTCCCGGTATA
*il17a/f3*	ZDB-GENE-041001-192	TCAAAGAAAGACAGCTTGGGT	AACAGAAGTTGTGTATGTCCAA
*ccl19a.1*	ZDB-GENE-060526-181	CCCACGTGATGCTGTAATATT	AGCGTCTCTCGATGAACCTT
*ccl20a.3*	ZDB-GENE-081022-193	AGCTGTGTCGTGTTGCAGAA	CCGTTTGTGTGGAATATGACA
*b-actin (EPC cells)*	*Pimephales promelas* gene	GATGACGCAGATCATGTTCGAG	CCGCAAGATTCCATACCAAGGAAGG
			
**Construction primers**			
*rps11* standard	ZDB-GENE-040426-2701	CCCAGAGAAGCTATTGATGGC	TCACATCCCTGAAGCATGGG
hACE2NotStart3		TATAGCGGCCGCGGGGACGATGTCAAGCTCTTCCT
ACE2EndNot3		TATAGCGGCCGCAAAAGGAGGTCTGAACATCA
hAce2.2ANot		AATTGCGGCCGCAGGGCCCAGGGTTGGACTCGACGTCTCCCGCAAGCTTAAGAAGGTCAAAATTCAACAGCTGAGATCTAAAGGAGGTCTGAACATCAT	

### Viruses

The main SARS-CoV-2 stock used (BetaCoV/France/IDF0372/2020 strain) was propagated twice in Vero-E6 cells and is a kind gift from the National Reference Centre for Respiratory Viruses at Institut Pasteur, Paris, headed by Dr Sylvie van der Werf; this strain was isolated from a human sample provided by Drs. Xavier Lescure and Yazdan Yazdanpanah from the Bichat Hospital, Paris. To generate concentrated virus, Vero-E6 cells were infected with virus at an MOI of 0.01 PFU/cell in DMEM/2% FBS and incubated for 72 h at 37°C, 5% CO_2_. At this point, the cell culture supernatant was harvested, clarified, and concentrated using Amicon Ultra-15 Centrifugal units 30K (Merck Millipore, Burlington, MA, USA). Virus titers were quantified by plaque assay in Vero-E6.

The variant strains used were also supplied by the National Reference Centre for Respiratory Viruses at Institut Pasteur and were used directly without further propagation. The G-clade (BetaCoV/France/GE1973/2020; 3 × 10^7^ PFU/ml), alpha (hCoV-19/France/IDF-IPP11324i/2020; 6.75 × 10^7^ PFU/ml), beta (hCoV-19/France/PDL-IPP01065i/2021; 1.75 × 10^8^ PFU/ml), and gamma (hCoV-19/French Guiana/IPP03772i/2021; 5.53 × 10^7^ PFU/ml) variants were isolated from human samples provided respectively by Dr. Laurent Andreoletti, from Robert Debré Hospital, Reims, France; Dr. Foissaud, HIA Percy, France; Dr. Besson J. from Bioliance Laboratory, France; and Dr. Rousset, Institut Pasteur, Cayenne, French Guiana.

The SINV-GFP virus corresponds to the SINV-eGFP/2A strain described in Boucontet et al. (2018) and was used as a BHK cell supernatant at 2 × 10^7^ PFU/ml. The eGFP sequence is inserted with self-cleaving sequences between the capsid and envelope genes of SINV.

### Bath Exposure

Bath exposures were conducted in a 12-well plate with 4 larvae per well in 2 ml of water plus PTU. 2-dpf embryos were manually dechorionated previously. 10 µl of SARS-CoV-2 suspension 2 (either freshly thawed or heat-inactivated for 5 min at 70°C) was added to each well and gently mixed, then the plates were incubated at 32°C. After a given incubation time, larvae were deeply anesthetized with 0.4 mg/ml tricaine (MS222, Sigma-Aldrich), rinsed twice in 10 ml of water, transferred individually into tubes, and after removal of almost all water, lysed in 320 µl of RLT buffer (Qiagen, Hilden, Germany) supplemented with 1% β-mercaptoethanol (Sigma-Aldrich).

## Microinjection

SARS-CoV-2 microinjections are carried out under a microbiological safety hood inside a BSL3 laboratory, in which a camera-fitted macroscope (DMS1000, Leica, Wetzlar, Germany) with a transilluminated base is installed, as in (Van Dycke et al., 2019). Borosilicate glass capillaries are loaded with a concentrated SARS-CoV-2 suspension previously colored by the addition of 10% (V/V) of 0.5% phenol red in PBS (Sigma), then connected to a FemtoJet 4i microinjector (Eppendorf, Framingham, MA, USA). Otherwise, the procedure was similar to the one detailed in Levraud et al. (2008). After breakage of the capillary tip, pressure was adjusted to obtain droplets with a diameter of ~0.13 mm. Larvae at the desired developmental stage were anesthetized with 0.2 mg/ml tricaine and positioned and oriented in the groove molded in agarose of an injection plate overlaid with water containing tricaine. Using a micromanipulator, the capillary was then inserted at the desired site and two pulses performed to inject approximately 2 nl. Proper injection is ascertained visually with the help of phenol red staining; otherwise, the larva is discarded. A picture of the injected larva is taken with the camera, and it is then rinsed by transfer inside a water-filled Petri dish and immediately transferred to its individual well in a 24-well plate, containing 1 ml of water with PTU. Larvae are then incubated at 32°C (actual temperatures measured inside the incubator ranged from 31.6°C to 33.2°C). At daily intervals, all larvae were anesthetized by addition of a drop of 4 mg/ml tricaine into each well and a snapshot was taken. A randomized subset of larvae was then transferred to tubes and individually lysed in 320 µl of RLT buffer + 1% β-mercaptoethanol. Water with tricaine was then removed from the remaining wells, replaced with 1 ml of freshwater with PTU, and the plate returned to the incubator.

SINV injections were performed in a BSL2 laboratory as described in Passoni et al. (2017).

### Lysis, RNA Extraction, and RT-qPCR of Larvae

After addition of RLT buffer, larvae were dissociated by 5 up- and-down-pipetting movements. Tubes may then be frozen at -80°C for a few days. Before export from the BSL3 laboratory, RLT lysates were incubated at 70°C for 5 min to ensure complete virus inactivation (preliminary tests confirmed that this had a negligible impact on qRT-PCR results). Total RNA was then extracted with an RNeasy Mini Kit (Qiagen) without the DNase treatment step and a final elution with 30 µl of water.

RT was performed on 6 µl of eluted RNA using MMLV reverse transcriptase (Invitrogen, Carlsbad, CA, USA) with either a dT_17_ primer (for polyadenylated transcripts) or the SgleadSARSCoV2-F primer (for negative-strand viral transcripts) (Wölfel et al., 2020) (**Table 2**). cDNA was diluted with water to a final volume of 100 µl, of which 5 µl was used as a template for each qPCR assay.

Real-time qPCR was performed with an ABI7300 (Applied Biosystems, Foster City, CA, USA). Quantitation of sense or antisense viral *N* transcripts was performed by a TaqMan probe assay, using the primer–probe mix from the 2019-nCoV RUO kit (IDT, Coralville, IA, USA) with the iTaq Universal Probes One-Step kit (Bio-Rad, Hercules, CA, USA). The 2019-nCoV_N_Positive Control plasmid (IDT) was used as a standard for absolute quantification. Quantification of zebrafish transcripts was performed using a SYBR assay using the Takyon SYBR Blue MasterMix (Eurogentec, Seraing, Belgium) with primer pairs listed in **Table 2**. These primers typically span exon boundaries to avoid amplification of contaminating genomic DNA. For absolute quantification of the housekeeping gene *rps11*, a standard was produced by PCR using primers to amplify a fragment including the whole open-reading frame, which was gel-purified and quantified by spectrophotometry. Ratios of other transcripts to *rps11* were estimated by the 2^ΔCt^ method.

### Morpholino and Plasmid Injection in Eggs

Morpholino antisense oligonucleotides (Gene Tools, Philomath, OR, USA) were injected (1 nl volume) in the cell or yolk of AB embryos at the one- to two-cell stage as described (Levraud et al., 2008). crfb1 splice morpholino (2 ng, CGCCAAGATCATACCTGTAAAGTAA) was injected together with crfb2 splice morpholino (2 ng, CTATGAA TCCTCACCTAGGGTAAAC), knocking down all type I IFN receptors (Aggad et al., 2009). Control morphants were injected with 4 ng control morpholino, with no known target (GAAAGCATGGCATCTGGAT CATCGA).

Expression plasmids, produced using an endotoxin-free kit (Macherey-Nagel, Düren, Germany), were co-injected with the I-SceI meganuclease (Grabher et al., 2004). Briefly, 12.5 µl of plasmid is mixed with 1.5 µl of CutSmart Buffer and 1 µl of I-SceI (New England Biolabs, Ipswich, MA, USA) and incubated at room temperature for 5 min before being put on ice until injection of 1 nl inside the cell of AB embryos at the one-cell stage.

### Live Fluorescence Imaging

SINV-GFP-infected or hACE2-mCherry-expressing larvae were imaged with an EVOS FL Auto microscope (Thermo Fisher Scientific, Waltham, MA, USA) using a 2× planachromatic objective (numerical aperture, 0.06), allowing capture of the entire larva in a field. Transmitted light and fluorescence (GFP or Texas Red cube) images were taken. They were further processed (superposition of channels, rotation, crop, and fluorescence intensity measure) using Fiji. Mean background fluorescence of uninjected control animals was subtracted from the measured signal to obtain the specific fluorescence.

### Flow Cytometry

Pools of 10 larvae were dissociated by a combination of mechanical trituration (repeated pipetting) and enzymatic treatment at 30°C, first with 200 µl of 0.25% Trypsin-EDTA (Gibco, Grand Island, NY, USA) for 10 min, and 10 more min after addition of 10% sheep serum, CaCl_2_ to 2 µM, and 1 µl of 5 mM collagenase (C9891, Sigma). Cell suspensions were then washed with PBS 1×, pelleted, resuspended in PBS, and filtered on a 40-µm mesh. Dead cells were labeled with SYTOX AADvanced (Thermo Fisher). Cell suspensions were acquired on an Attune NxT flow cytometer (Thermo Fisher) with blue and yellow lasers, and data analyzed with FlowJo (Ashland, OR, USA).

### Cell Culture

The *Epithelioma papulosum cyprini* cell line (EPC) was maintained in Leibovitz’s 15 media (L-15, Gibco) supplemented with 10% fetal bovine serum (FBS, Gibco), 100 µg/l penicillin, and 100 µg/ml streptomycin. EPC cells were cultured at 32 °C without CO_2_.

BHK-21 cells were maintained in DMEM (Gibco) supplemented with 5% FBS and 2 mM L-glutamine and maintained at 37°C with 5% CO_2_.

### Human ACE2-Expressing Constructs

The hACE2 ORF was amplified from clone IOH80645 (Thermo Fisher, GenBank NM_021804.2) using primers hACE2NotStart3 and hACE2EndNot3 (**Table 2**). The amplified PCR fragment was digested by NotI and inserted in the NotI site of the Tol2S263C:mC-F vector between the promoter of the zebrafish ubiquitous ribosomal protein RPS26 encoded by chromosome 3, and the mCherry ORF. In this construct, the RPS26 promoter drives the expression of a hACE2 protein fused at its C-term with farnesylated mCherry. In order for the ORF to drive the expression of two separated proteins (hACE2 and mCherry-F), primers hACE2NotStart3 and hAce2.2ANot were used to amplify the hACE2 ORF from the IOH80645 clone. The amplified fragment was digested by NotI and cloned in the NotI site of Tol2S263C:mC-F between the promoter of the zebrafish ubiquitous ribosomal protein RPS26 encoded by chromosome 3, and the mCherry ORF. Maps and sequences of plasmids are available at https://doi.org/10.5281/zenodo.4672028.

For *in vitro* transfection of EPC cells, plasmid pcDNA3.1-hACE2 (Addgene, Watertown, MA, USA, #1786) was directly used along plasmid pmEGFP-N1 (Chen and Reich, 2010).

### Cell Transfection

EPC cells were electroporated with the Neon transfection system (Invitrogen). Briefly, EPC cells were trypsinized and resuspended in L15 media supplemented with FBS and antibiotics. Cells were counted and centrifuged at 2,000 rpm for 5 min. 0.8 × 10^6^ cells per condition were resuspended in 80 µl of L15 without phenol red with 2.4 µg of each plasmid. Cells were electroporated using 10-µl neon tips with 1 pulse of 1,700 V during 20 ms. Electroporated cells were plated in a 6-well plate in L15 + FBS + antibiotics and incubated 3 days at 32°C before experiment.

BHK21 were transfected with Lipofectamine 2000 (Invitrogen). Briefly, cells at 80% confluence in 12-well plates were incubated with 750 ng of plasmid and 1 µl of Lipofectamine in Opti-MEM (Gibco). Transfection efficiency was checked at 1 day post-transfection.

### Cell Infection and RT-qPCR

Transfected EPC cells were transferred to BSL3 laboratory for infection with SARS-CoV-2. Cells were rinsed with L15 media + FBS + antibiotics and incubated 5 min at 32°C. Cells were infected at MOI 0.1 with virus diluted in L15 media + FBS + antibiotics and incubated at 32°C during 1 h with agitation. After incubation, L15 media + 10% FBS + antibiotics was added and cells were incubated 2 days at 32°C or processed directly for RNA extraction.

Transfected BHK21 cells were transferred to BSL3 laboratory for infection with SARS-CoV-2. Cells were rinsed with DMEM + 5% FBS and incubated for 5 min at 37°C + 5% CO_2_. Cells were infected with virus at MOI 0.1 diluted in DMEM + 5% FBS and incubated at 37°C + 5% CO_2_ during 1 h with agitation. After incubation, DMEM + 5% FBS was added and cells were incubated 2 days at 32°C or processed directly for RNA extraction.

Before RNA extraction, culture medium was removed and cells were rinsed once with PBS. Extraction of total RNA was performed using TRI Reagent (Sigma) following the manufacturer’s recommendations. Total RNA was resuspended in 100 µl of RNase-free water.

Reverse transcription was performed on 5 µl of RNA suspension using the QuantiTect Reverse Transcription Kit (Qiagen) with either the Qiagen RT Primer Mix or the SgleadSARSCoV2-F primer (for negative-strand viral transcripts) (Wölfel et al., 2020). cDNA was diluted with water to a final volume of 50 µl, of which 2.5 µl was used as a template for each qPCR assay.

Real-time qPCR was performed with a RealPlex 2 (Eppendorf). Quantitation of viral N transcripts was performed by a TaqMan probe assay, using the primer–probe mix from the 2019-nCoV RUO kit (IDT) with the iTaq Universal Probes kit (Bio-Rad). Quantitation of actin transcripts was performed by a SYBR Green assay, using primers specific for fathead minnow β-actin (**Table 2**) with iTaq Universal SYBR Green Supermix (Bio-Rad).

### Immunohistochemistry

Whole-mount immunohistochemistry of larvae was performed essentially as described in Palha et al. (2013) and Santos et al. (2018). For COV2-N detection, additional treatment with glycine 0.3 M in PBST (30 min at RT) and heat-induced antigen retrieval (HIER) were performed. HIER treatment was performed in 150 mM Tris–HCl, pH 9.0, at 70°C for 15 min. Primary Ab antibodies used for this labeling were mouse anti-SARS-CoV-2 nucleoprotein (Sino Biological, Beijing, China, 40143-MM05, 1:100) and rabbit anti-GFAP (GeneTex, Irvine, CA, USA, GTX128741, 1:100). As secondary Ab antibodies used were goat anti-mouse F(ab)′2 Alexa Fluor 488 (Molecular Probes, Eugene, OR, USA, A11017, 1:300) and goat anti-rabbit Cy3 (Jackson Laboratories, Bar Harbor, ME, USA, 111-166-003, 1:300). Furthermore, to label the nuclei NucRed Live 647 (Thermo Fisher, R37106, 4 drops for mL for 45 min) was used. For hACE2 detection, staining was performed sequentially since both the primary Ab for ACE2 and the secondary Ab for mCherry were from goat. Primary staining for ACE2 (goat anti-ACE2, AF933, R&D Systems, Minneapolis, MN, USA, 4 µg/ml) was performed first, followed by its secondary staining (donkey anti-goat Ig Alexa 488, A100555, Invitrogen, 1:300), then primary staining for mCherry (rabbit anti-DsRed, 632393, Clontech, Mountain View, CA, USA, 1:300) and secondary staining (goat anti-rabbit Ig Cy3, 111-166-003, 1:300). Nuclei were labeled with 2 µg/ml Hoechst 33342 (Invitrogen). To label membranes, we used DilC18(3) (Thermo Fisher Scientific, D282) staining at 1 µM working solution for 24 h at 4°C, followed by 6 × 45-min washes in PBST.

Afterward, IHC larvae were conserved in 80% glycerol until acquisition. For acquisition of N-CoV-2, the larvae were mounted in 2% agarose in 80% glycerol singularly in a glass-bottom 8-well slide (ibidi, Gräfelfing, Germany, 80827). Images were acquired using an inverted confocal microscope Leica SP8 using a ×10 objective zoomed 1.25× (PL FLUOTAR ×10/0.30 DRY) and ×20 immersion objective (HC PL APO CS2 ×20/0.75 multi-IMM). For both magnification, the bidirectional resonant scanning method was used and images were deconvolved using Leica Lightning Plug-in. For acquisition of hACE2, images were acquired on an upright Leica SPE confocal microscope using a ×40 oil objective (numerical aperture, 1.15).

For IHC of *in vitro* transfected cells, EPC were cultured in a 6-well plate containing sterilized coverslips. At 3 days post-transfection, culture media were removed, and cells were rinsed with PBS once. Cells were fixed overnight at 4°C with 4% methanol-free formaldehyde (Sigma) in PBS. Formaldehyde was removed, and cells were rinsed twice with PBS and kept at 4°C in PBS + 0.05% sodium azide. Fixed cells were rinsed 3 times in PBS. Cells were then permeabilized and blocked with PBS + 0.3% Triton X-100 + 10% horse serum during 45 min at RT. Cells were stained for 1 h at RT with a goat polyclonal anti-human ACE2 (AF933, R&D Systems) diluted at 3 µg/ml in PBS + 0.3% Triton X-100 + 1% horse serum + 1% BSA + 0.01% sodium azide. Cells were then rinsed and stained during 1 h at RT with Alexa 647 anti-goat diluted at 1/500 in PBS + 0.3% Triton X-100 + 1% horse serum + 1% BSA + 0.01% sodium azide. After 3 rinses with PBS, cells were incubated for 1 h at RT with DAPI diluted at 2.5 µg/ml in PBS. After 3 rinses in PBS, coverslips were mounted on slides with Fluoromount-G (Thermo Fisher Scientific).

Transfection efficiency was checked at 3 days post-transfection using a Zeiss Axio Observer Z1 widefield microscope with a ×10/NA 0.25 objective. The phase and GFP channel were acquired on a field of view of 5. Confocal acquisition of immunostained EPC cells was performed on a Leica SP8 upright microscope using a ×25/NA 0.95 coverslip-corrected objective. Endogenous GFP and Alexa 647 were excited with 488 and 638 nm, respectively, and detected with PMT. Fiji was used to adjust the brightness and contrast of confocal images of immunostained EPC cells. Transfection efficiency was quantified using Fiji by manually counting total cells and GFP-expressing cells, respectively.

### Statistical Analysis

Analyses were performed with GraphPad Prism. Methods used are indicated in Figure legends. Normality/log-normality tests of data distribution were performed to decide the most appropriate assays.

## Data Availability Statement

The raw data supporting the conclusions of this article will be made available by the authors, without undue reservation.

## Author Contributions

J-PL, PB, IS, and MV designed the study, which was coordinated by J-PL. VR generated the concentrated SARS-CoV-2 virus and supervised early BSL3 work in IP. BdC supervised BSL3 work in INRAE. J-PL and VL performed 1-cell injections and SARS-CoV-2 microinjections in the BSL3 lab. VL performed SINV injections, WIHC, and fluorescence imaging. MF performed *in vitro* work. J-PL, LB, VL, and MF performed qRT-PCR assays. GL generated the overexpression plasmids. J-PL wrote the manuscript with input from all authors. All authors contributed to the article and approved the submitted version.

## Funding

This work was supported by the “Urgence COVID-19” fundraising campaign of Institut Pasteur, a dedicated grant co-funded by Institut Pasteur and Institut du Cerveau-Paris Brain Institute, the “ZebraCorona” grant from the exceptional research program call of Université Paris-Saclay, the MUSE-University of Montpellier COVID program, the Agence Nationale de la Recherche (Grant ANR-16-CE20-0002-03 « fish-RNAvax »), and the “ImageInLife” Innovative Training Network funded by European Community’s Horizon 2020 Marie-Curie Program under grant agreement no. 721537.

## Conflict of Interest

The authors declare that the research was conducted in the absence of any commercial or financial relationships that could be construed as a potential conflict of interest.

## Publisher’s Note

All claims expressed in this article are solely those of the authors and do not necessarily represent those of their affiliated organizations, or those of the publisher, the editors and the reviewers. Any product that may be evaluated in this article, or claim that may be made by its manufacturer, is not guaranteed or endorsed by the publisher.

## Supplementary Material

Supplementary Video S1 3D reconstruction of a larva inoculated with SARS-CoV-2 in the swim bladder after immunodetection of CoV-2 nucleoprotein (green) and GFAP (red). Nuclei are shown in blue. Related to (left-most panel).

Supplementary Video S2 3D reconstruction of the swim bladder area of a larva inoculated with SARS-CoV-2 in the swim bladder after immunodetection of CoV-2 nucleoprotein (magenta) with nuclei are shown in cyan. Related to **Figure S3C** (10^th^ panel).

Supplementary Video S3 Confocal Z-stack of the same larva as in **Supplementary Video S2**. Yellow arrows point to some cells with intimate contact of the two labels strongly suggesting presence of the SARS-CoV-2 nucleoprotein inside the cell cytosol.

Supplementary Video S4 Confocal Z-stacks of the swim bladder area of a larva inoculated with SARS-CoV-2 in the swim bladder after immunodetection of CoV-2 nucleoprotein (green) with membranes labelled by DiI (red) and nuclei shown in blue. Related to Panels E-H of **Figure 5**, showing 15 confocal planes.

Supplementary Video S5 Confocal Z-stack of the swim bladder area of a larva inoculated with SARS-CoV-2 in the swim bladder after immunodetection of CoV-2 nucleoprotein (green) with membranes labelled by DiI (red) and nuclei shown in blue. Related to **Figure 5I**.
