# Supplementary figures and images for: Exploring Zebrafish Larvae as a COVID-19 Model: Probable Abortive SARS-CoV-2 Replication in the Swim Bladder
Source: Front Cell Infect Microbiol. 2022 Mar 11;12:790851. doi: 10.3389/fcimb.2022.790851 (PMC8963489; doi:10.3389/fcimb.2022.790851)

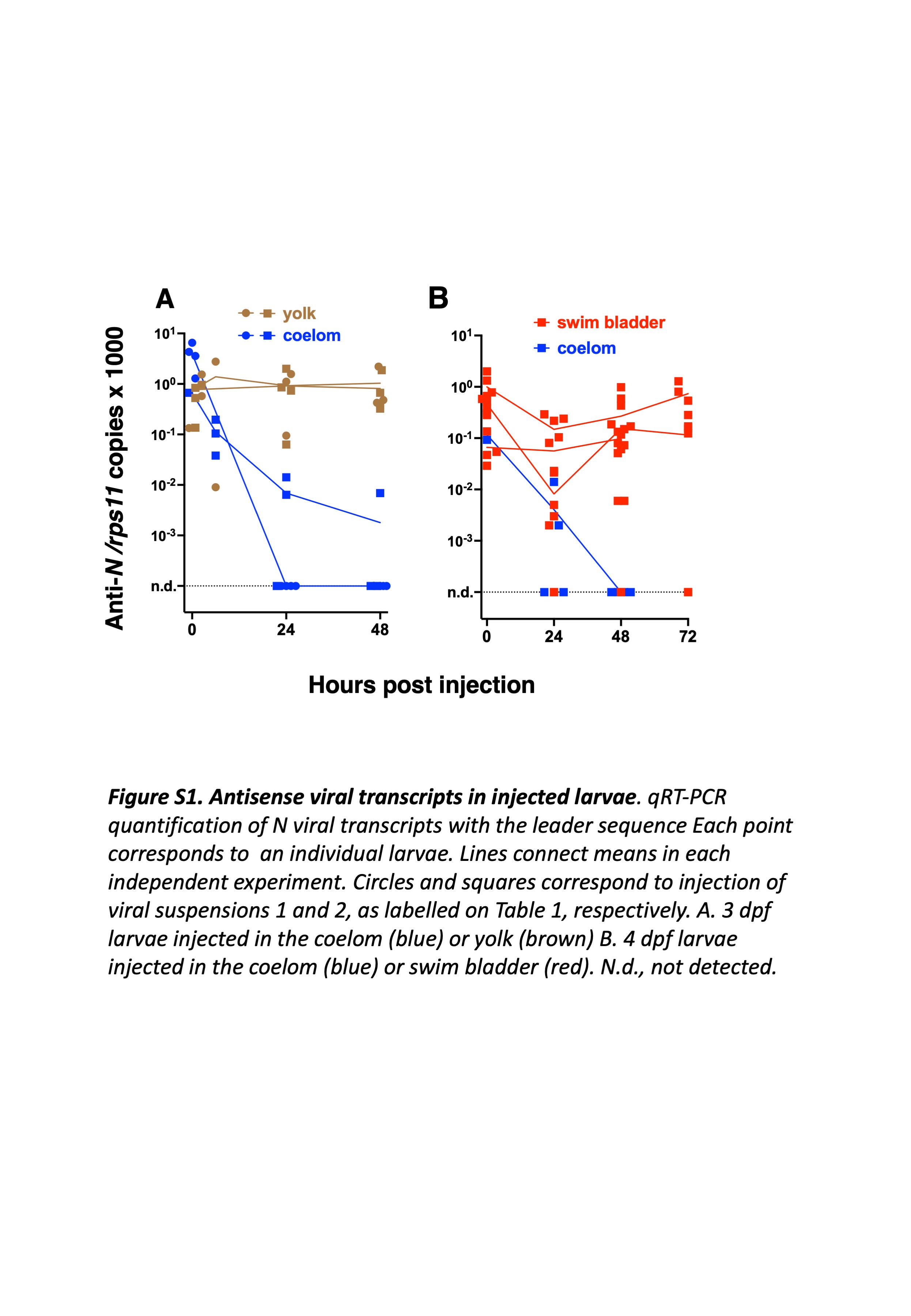

Supplement: Supplementary file 6 [file Image_1.jpeg]

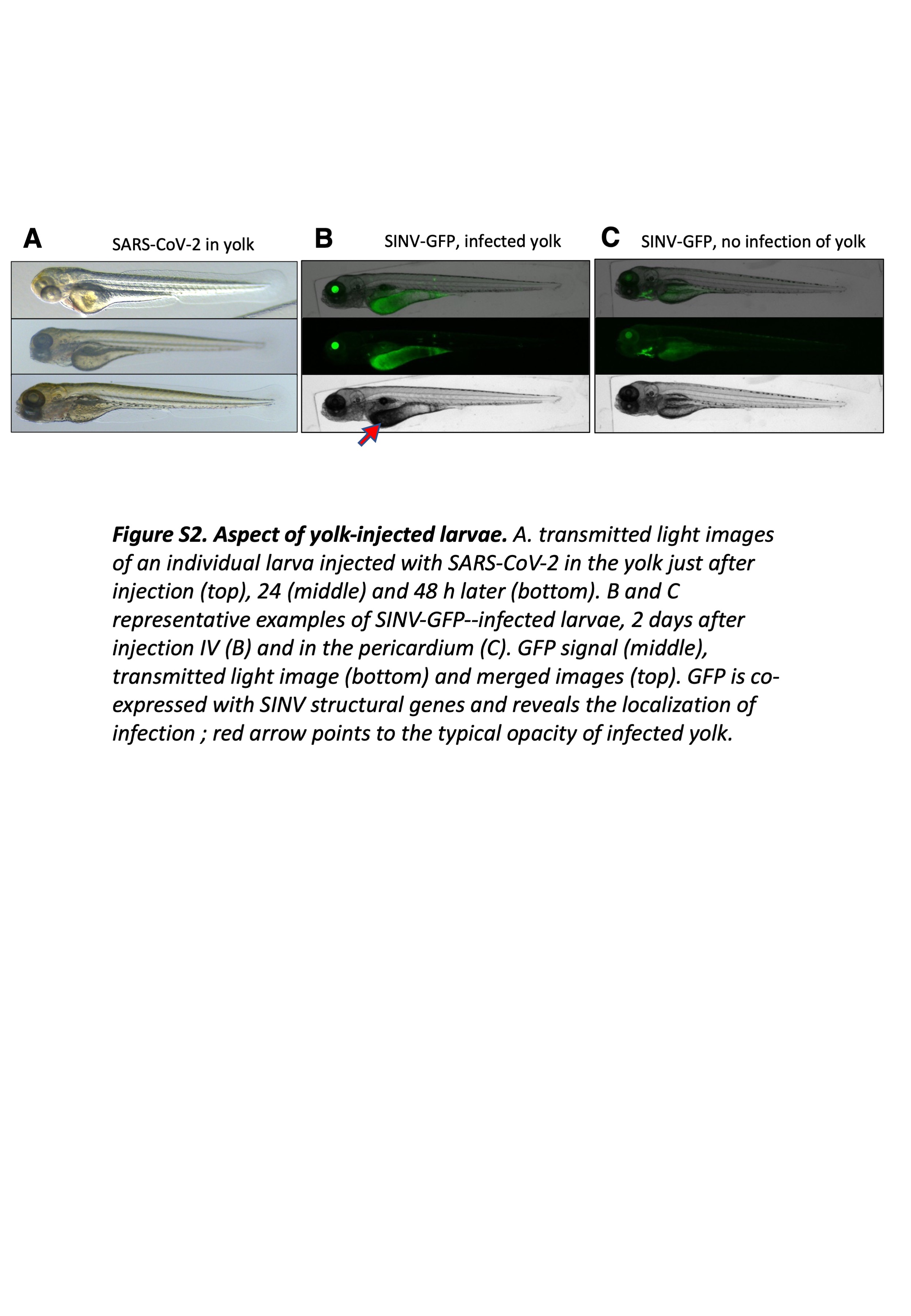

Supplement: Supplementary file 7 [file Image_2.jpeg]

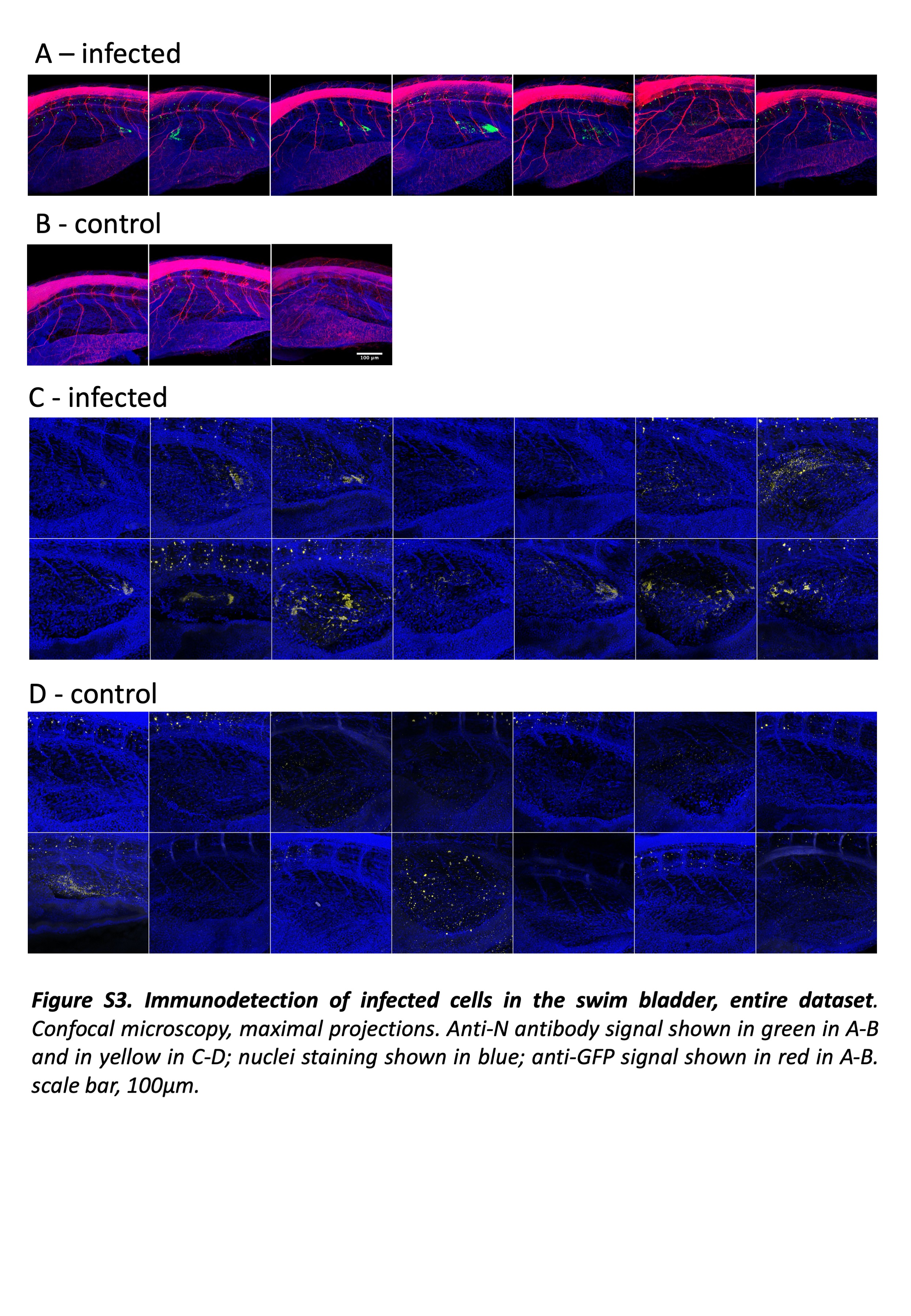

Supplement: Supplementary file 8 [file Image_3.jpeg]

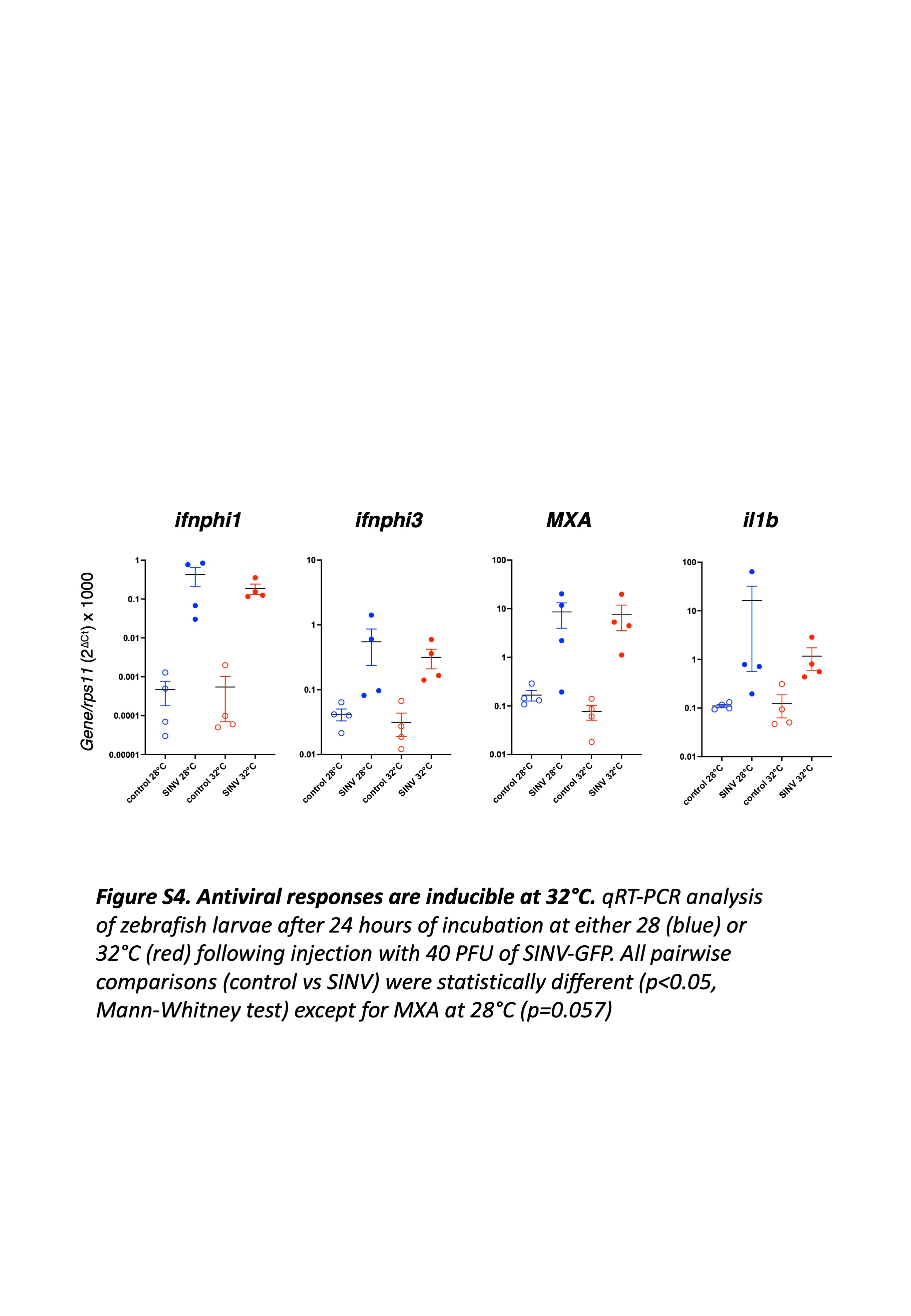

Supplement: Supplementary file 9 [file Image_4.jpeg]
